# A Systematic Review: Quercetin—Secondary Metabolite of the Flavonol Class, with Multiple Health Benefits and Low Bioavailability

**DOI:** 10.3390/ijms252212091

**Published:** 2024-11-11

**Authors:** Olimpia-Daniela Frenț, Liana Stefan, Claudia Mona Morgovan, Narcis Duteanu, Ioana Lavinia Dejeu, Eleonora Marian, Laura Vicaș, Felicia Manole

**Affiliations:** 1 Department of Pharmacy, Faculty of Medicine and Pharmacy, University of Oradea, No. 29 Nicolae Jiga Street, 410028 Oradea, Romania; daniela.olimpia@yahoo.com (O.-D.F.); marian_eleonora@yahoo.com (E.M.); laura.vicas@gmail.com (L.V.); 2 Department of Surgical Discipline, Faculty of Medicine and Pharmacy, University of Oradea, 410087 Oradea, Romania; 3 Department of Chemistry, Faculty of Informatics and Sciences, University of Oradea, No 1 University Street, 410087 Oradea, Romania; 4 Faculty of Chemical Engineering, Biotechnologies, and Environmental Protection, Politehnica University of Timisoara, No. 2 Victoriei Square, 300006 Timişoara, Romania; 5 National Institute of Research and Development for Electrochemistry and Condensed Matter, 144 Dr. A. P. Podeanu, 300569 Timisoara, Romania

**Keywords:** secondary metabolites, flavonoids, diseases, quercetin, health benefits, bioavailability

## Abstract

The main goal of this systematic review on the flavonol class secondary metabolite quercetin is to evaluate and summarize the existing research on quercetin’s potential health benefits, therapeutic properties, and effectiveness in disease prevention and treatment. In addition to evaluating quercetin’s potential for drug development with fewer side effects and lower toxicity, this type of review attempts to collect scientific evidence addressing quercetin’s roles as an antioxidant, anti-inflammatory, antibacterial, and anticancer agent. In the first part, we analyze various flavonoid compounds, focusing on their chemical structure, classification, and natural sources. We highlight their most recent biological activities as reported in the literature. Among these compounds, we pay special attention to quercetin, detailing its chemical structure, physicochemical properties, and process of biosynthesis in plants. We also present natural sources of quercetin and emphasize its health benefits, such as its antioxidant and anti-inflammatory effects. Additionally, we discuss methods to enhance its bioavailability, analyzing the latest and most effective delivery systems based on quercetin.

## 1. Introduction

Plants are sensitive organisms that can be exposed during their lifetime to a series of biotic and abiotic stresses. To adapt to environmental conditions and overcome stress conditions, plants synthesize a range of secondary metabolites [1,2,3]. These compounds perform essential physiological and biochemical functions [4]. Among these secondary plant-synthetized metabolites, flavonoids represent one of the largest and most well-investigated classes [1,5].

Flavonoids play several important roles in plants, including controlling growth and differentiation, as well as producing colors (pigments), texture, and taste. They possess a diverse range of structural features that contribute to their antioxidant and UV-protective properties in plants [6,7]. The specific chemical structure of flavonoids, characterized by two aromatic rings connected by a three-carbon bridge, gives rise to a wide range of functional groups and derivatives that are responsible for the diverse bioactivities exhibited by these plant metabolites [8]. The presence of phenolic hydroxyl groups on the flavonoid backbone allows for the donation of hydrogen atoms or electrons, which can stabilize free radicals and reactive oxygen species, thereby conferring strong antioxidant properties [9]. Additionally, the conjugated double bonds within the flavonoid structure can absorb UV radiation, protecting plant tissues from harmful solar exposure. By combining these properties, flavonoids play a critical role in shielding plants from oxidative damage while also participating in essential biological functions like growth regulation and pigment production [10].

Due to their potentially beneficial role in human metabolism, flavonoids are currently considered indispensable components in various nutraceutical, pharmaceutical, medicinal, and cosmetic applications. These bioactive compounds can protect the human body from the onset of degenerative conditions associated with oxidative stress including cancer, osteoporosis, diabetes mellitus, asthma, neurodegenerative diseases, Parkinson’s disease, dementia, and cardiovascular and inflammatory diseases [1,11,12,13,14,15].

The flavonoid quercetin, which belongs to the flavonol subclass [16], is the most abundant and widespread flavonoid compound in the plant kingdom. It is present in various array of sources, including peels, bark, vegetables (onions, broccoli, peppers, lovage, dill, cabbage), fruits (apples, aronia, cranberries), seeds (coriander, walnuts), red wine, green tea, etc. [6,17,18,19,20]. Chemically designated as 3,5,7-trihydroxy-2-(3,4-dihydroxyphenyl)-4H-chromen-4-one, quercetin serves as a pharmacologically active ingredient in numerous dietary supplements and in antioxidant and antiallergic drugs approved by the FDA (U.S. Food and Drug Administration) [16,21]. The most important pharmacological properties of quercetin reported in the literature are antioxidant, antidiabetic, anticancer, antitumor, anti-inflammatory, antiallergic, antihypertensive, antidepressant, cataract-preventive, and antimicrobial effects [22,23,24]. In addition, quercetin has demonstrated efficacy in inhibiting the progression of various cancers, including those affecting the breast, cervix, lung, colon, prostate, and liver [25]. These multiple benefits position quercetin as an important valuable nutraceutical ingredient, underscoring its substantial therapeutic potential within both the food and pharmaceutical sectors [16,22]. 

Quercetin’s low oral bioavailability has a significant impact on its pharmacological application. Despite its numerous health benefits, several challenges limit its utilization in the pharmaceutical sector. Low solubility in water limits its ability to traverse the intestinal mucus barrier and be absorbed by the enterocytes of the digestive tract. This phenomenon may be attributed to the hydrophobic nature of the phenyl rings, combined with the polar characteristics imparted by the hydroxyl groups present in its molecular structure [16,22,26]. Low aqueous solubility (1 μg/mL), diminished intrinsic activity (<10%), a high metabolic rate (>40%), rapid clearance from the organism (<1 h), and the formation of inactive metabolic products may adversely affect its efficacy and restrict its clinical application [27]. Research involving healthy subjects has shown that only 3–17% of the administered dose of quercetin is absorbed, with a slightly higher absorption rate of approximately 20% observed in animal studies [28]. In the context of recent advancements in biotechnology, the scientific community has tried to overcome the challenges associated with the poor oral bioavailability of quercetin through various encapsulation techniques [22]. By encapsulating quercetin in liposomal delivery systems, it was observed that it had a much greater ability to scavenge free radicals compared to non-encapsulated quercetin. This improvement can be attributed to the prolonged duration of action and increased solubility in water [29]. Furthermore, research conducted by Gulec K. and David M. indicated that incorporating quercetin into inclusion complexes such as methyl-β-cyclodextrin, resulted in a remarkable 254-fold increase in water solubility, along with a 10% enhancement in antioxidant activity relative to quercetin alone [30]. Abouaitah et al. included quercetin in mesoporous silica nanoparticles (MSNs) conjugated with folic acid to enhance absorption and facilitate targeted release into tumor cells. The findings indicated an increase in the biocompatibility of quercetin, while the MSN formulation demonstrated favorable characteristics, including a reduced particle size and heightened absorptive capability [31].

The main purpose of this review was to present the main classes of flavonoid compounds, with a focus on quercetin. Thus, we discussed the physicochemical properties, natural sources, and the biological activity associated with quercetin. In the end, we listed and described various delivery systems that can enhance the bioavailability of quercetin. In the context of pharmaceutical applications, it is imperative that quercetin delivery systems are both effective and capable of improving the pharmacokinetic profile, while also ensuring controlled transport and release within the body.

## 2. Materials and Methods

The systematic evaluation of this review was carried out by taking into account the Preferred Reporting Items for Systematic Reviews and Meta-Analysis (PRISMA).

### 2.1. Study Selection Criteria

The criteria for selecting scientific articles focused on the following aspects: the chemical structure and classification of flavonoid compounds; natural sources; physicochemical properties; and the health benefits of both flavonoid compounds and quercetin. Additionally, the selection included considerations of quercetin’s biosynthesis, metabolism, bioavailability, dosage, contraindications, and drug interactions. 

The criteria we considered for excluding certain articles were as follows: (i) articles not written in the English language; (ii) incomplete or briefly drafted articles; (iii) articles that focused more on quercetin derivatives rather than on quercetin itself; and (iv) articles that referenced other polyphenolic compounds more than flavonoids. 

### 2.2. Search Strategy and Data Extraction

All the data necessary to carry out this research were collected from works published between 1957 and 2024, without any constraints on the publication date of the articles [28]. The databases used to achieve the aforementioned goal were the Web of Science Core Collection, Pubmed, Scopus, and Google Scholar. To identify scientific articles published by 2024, keywords such as “quercetin” and “flavonoids” were used. After eliminating duplicates, we analyzed the articles based on the previously established inclusion criteria. After a thorough analysis, articles that met the exclusion criteria were removed. The remaining articles were deemed relevant articles and included in this systematic review.

## 3. Results and Discussion

### 3.1. Study Selection

In this systematic review, we aimed to identify and examine the most relevant original research or review articles published in the literature on flavonoid compounds and quercetin. 

**Figure 1:**
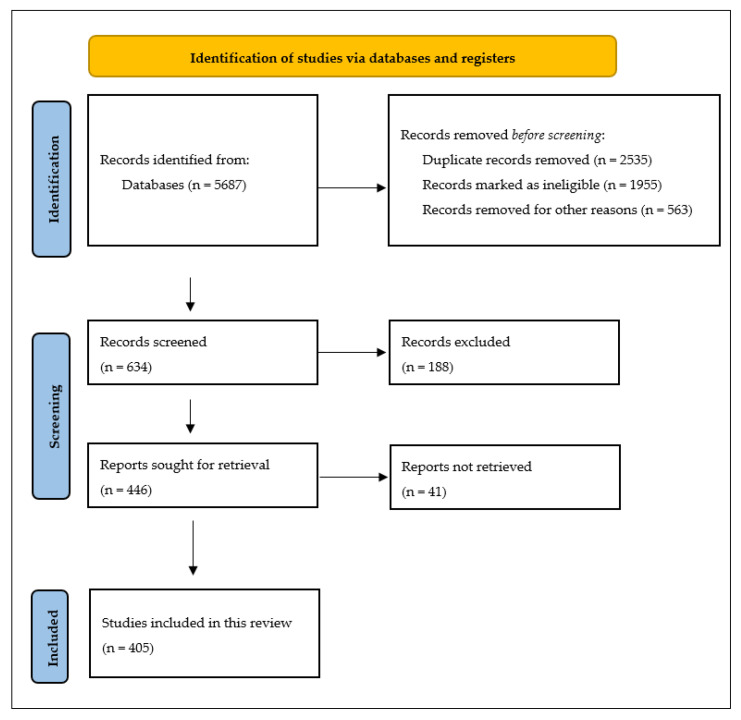
Prisma flow diagram for included studies.

The selection of studies was made following the Prisma flow diagram (see Figure 1).

As seen in this systematic review, we initially identified 5687 scientific articles. Following a preliminary evaluation, we removed 2535 duplicate studies found across various databases to eliminate redundancy. Furthermore, 1955 articles were excluded because they did not meet the eligibility and inclusion criteria, as they were unrelated to the research topic. Another 563 studies were excluded for other reasons, such as restricted access (available only for a fee) or being outside the designated field of research [32].

A total of 634 articles advanced to the screening stage, where their abstracts were reviewed. Of these, 188 studies that did not meet this study’s objectives were excluded. The remaining 446 articles were read in full, and after assessing their eligibility, 41 were excluded. Ultimately, 405 articles were included in this systematic review.

Notably, we observed that 279 of the included articles were published within the last four years, suggesting a growing interest in the study of flavonoids and quercetin.

### 3.2. Flavonoids—The Most Common Secondary Metabolites in the Plant Kingdom

#### 3.2.1. Structure and Classification of Flavonoid Compounds

**Figure 2:**
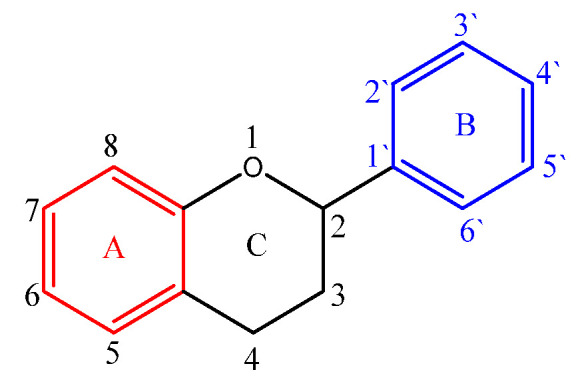
Basic chemical structure of flavonoids (prepared with Biorender, https://www.biorender.com/).

Flavonoids have a diphenyl propane base structure (C₆-C₃-C₆), as illustrated in Figure 2. This structure comprises 15 carbon atoms arranged into two aromatic rings, A and B. These rings are connected by three carbon atoms, which may form part of a third heterocyclic ring (C) [18,33,34,35].

The numbering of carbon atoms in rings A and C is completed using ordinary numbers, while in ring B, prime numbers are used, except for chalcones and some derivatives of isoflavones. The heterocyclic C ring can be either a pyran (as found in flavones or flavonols) or dihydro derivatives (as in flavanones or flavan-3-ol).

According to biogenesis, the A ring originates from a resorcin or phloroglycin molecule and can be hydroxylated at positions 3, 5, and 7. The B ring derives from shikimic acid and is hydroxylated at positions 2’, 3’, 4’, and 5’ [18,33].

**Figure 3:**
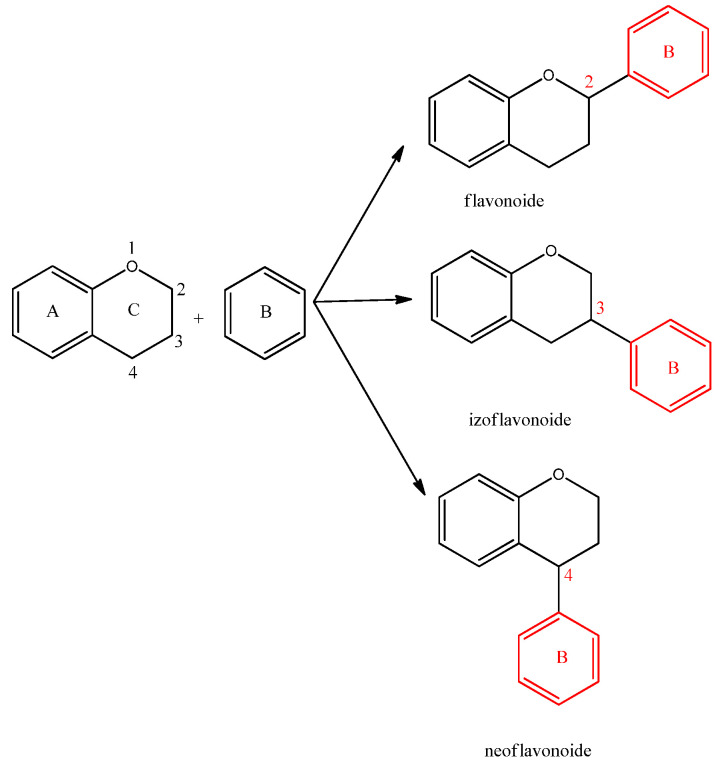
Main classes of flavonoids (prepared with Biorender).

Flavonoids can be divided into the following three classes based on the position of the benzenoid substituent relative to the benzopyranic substituent (C ring), as illustrated in Figure 3:

(a) Flavonoids (in which the benzenoid substituent is attached at position 2 of the C ring);
(b) Isoflavonoids (in which the benzenoid substituent is attached at position 3 of the C ring);
(c) Neoflavonoids (in which the benzenoid substituent is attached at position 4 of the C ring) [11].

Flavonols, unlike flavanones, exhibit a hydroxyl group at position 3 and a double bond between C_2_-C_3_ [33].

**Figure 4:**
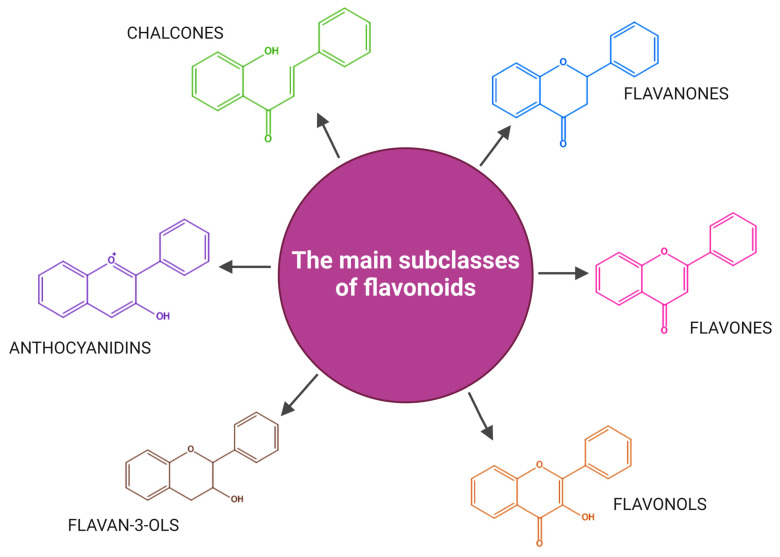
Classification of flavonoids according to the oxidation degree of molecules (prepared with Biorender software).

According to the number of hydroxyl or methyl groups attached to the aromatic ring B, the nature of the carbon atom of the C ring, and the degree of unsaturation of the heterocyclic ring C flavonoids can be classified into the following six subclasses, as illustrated in Figure 4 [11,18,34,35]:

(a) Flavones (present a double bond between the C_2_ and C_3_ positions and a ketone group at position 4 of the heterocyclic C ring) [35];
(b) Flavonols or 3-hydroxy-flavones (consist of two aromatic rings joined by a linear chain of three carbon atoms (C_2_, C_3,_ and C_4_)). In this chain, C_2_ and C_3_ are connected by a double bond, and a ketone group is present at C_4_ [36]. Flavonols may also have hydroxyl groups at specific positions: 5 and 7 of the A ring and position 3 of the C ring [11,37];
(c) Flavanones (the C ring is saturated, and the diphenyl propane conjugate system between the two aromatic rings is interrupted [11]);
(d) Flavan-3-ols or catechins (they are 3-hydroxyl derivatives of flavanones, with the hydroxyl group attached at position 3 of the heterocyclic ring C. Unlike flavonols, they do not have a double bond between the C₂ and C₃ positions [38]);
(e) Anthocyanins and anthocyanidins (differ from other flavonoids due to having two double bonds in the heterocyclic C rings. Anthocyanins are the glycosylated form of anthocyanidins, and differ in hydroxylation and methoxylation patterns of the B ring) [39];
(f) Chalcones (are characterized by the absence of the heterocyclic C ring in their basic diphenyl-propane structure [11]. Therefore, they can also be referred to as open-chain flavonoids [40]).

#### 3.2.2. Sources and Bioactivity of Flavonoid Compounds

Flavonoids in plants exist in several forms: free aglycone (which do not have attached sugar molecules), conjugated with sugar units (C-glycosylated or O-glycosylated), or methylated derivatives. Depending on the number of sugar units (glycosides) attached, flavonoids can be classified as monoglycosylated, diglycosylated, or polyglycosylated. The glycosyl group, which can be glucose, rhamnose, galactose, and arabinose, is linked at position 3 or 7 of the flavonoid’s basic structure [41]. Additionally, the nucleus of flavones and flavonols can have isoprene residues and free hydroxyl groups as substitution, or this site can be fully blocked by methylation. They can also be present in the form of esters of sulfuric acid or other organic acids [11].

##### Flavones

Flavones are an important subgroup of flavonoids that are widely found in leaves, flowers, and fruits. Most flavones in vegetables and fruits feature a hydroxyl group at position 5 of ring A. Hydroxylation can also occur at other positions, such as position 7 of ring A or at 3′ and 4′ of ring B, with this variation often depending on the taxonomic classification of certain vegetables or fruits [35]. Among flavones, apigenin, vitexin, and luteolin are notable for their widespread presence and significant health benefits. Apigenin, in its O-glycosylated form, is found in high concentrations in chamomile (up to 5320 mg/100 g of dry flowers) and parsley (1350 mg/100 g of dry leaves). Luteolin can be found in various plants, including fruits, and vegetables (green pepper, celery), as well as in medicinal herbs like chamomile. The C-glycosylated form of luteolin was found in the highest proportions in rooibos tea [42]. 

Apigenin, which is abundant in chamomile and parsley, has been shown to improve cognitive functions and memory in animal studies. Research suggests that it can regulate apoptosis, amyloidogenesis (the formation of amyloid plaques linked to Alzheimer’s disease), and the BDNF/TrkB signaling pathways [43]. By modulating these processes, apigenin has potential as a neuroprotective agent, particularly in the context of neurodegenerative diseases like Alzheimer’s. In addition to its neuroprotective properties, apigenin exhibits strong anti-inflammatory effects. Research indicates that it has demonstrated efficacy in reducing inflammation in mice with ulcerative colitis induced by dextran sodium sulfate (DSS). Apigenin facilitates the secretion of mucin, a key component that safeguards the intestinal lining, while concurrently promoting the production of anti-inflammatory cytokines such as IL-10, and inhibiting pro-inflammatory cytokines, including TNF-α, IL-1β, and IL-6. This action suggests that apigenin may hold promise for the treatment of inflammatory bowel diseases [44].

Vitexin, a compound found in foods like buckwheat, beans, and hawthorn, also demonstrates anti-inflammatory properties by inhibiting the production of pro-inflammatory cytokines. This suggests it may be beneficial in managing various inflammatory conditions [45]. In addition to its anti-inflammatory effects, vitexin has been shown to protect cells from oxidative stress, particularly in cancer-related situations. It exhibits anticancer properties by inducing apoptosis, or programmed cell death, in tumor cells, making it a promising candidate for cancer therapy. Vitexin can slow the progression of cancer by selectively targeting tumor cells for destruction while minimally affecting healthy cells. Moreover, studies conducted on rats indicate that vitexin has a hepatoprotective effect, meaning it protects the liver from damage caused by toxins like cadmium. It achieves this by reducing oxidative stress and inflammation in the liver [46].

Luteolin is found in various plant families including Asteraceae, Lamiaceae, Poaceae, Leguminosae, and Scrophulariaceae [47]. It has shown significant potential in combating Alzheimer’s disease by reducing neuroinflammation and preventing neuronal degeneration, both of which are major contributors to cognitive decline. By modulating these pathways, luteolin may help slow the progression of neurodegenerative disorders and improve overall brain function [48]. Additionally, luteolin has proven effective in alleviating intestinal mucositis induced by chemotherapy. This condition, often caused by drugs like irinotecan, can lead to weight loss and severe diarrhea. Luteolin has been shown to help alleviate these symptoms, making it a useful compound for improving the quality of life in patients undergoing chemotherapy [48]. The main sources of flavones and their biological activities are presented in Table 1. 

##### Flavonols

Flavonols play a crucial role in human health because of their various biological activities, which arise from their structural diversity. Their ability to undergo methylation, hydroxylation, and glycosylation leads to the formation of different flavonol types, such as quercetin, kaempferol, myricetin, rutin, morin, and isorhamnetin [88]. This diversity is reflected in the wide range of plant species that contain these compounds, including common vegetables like *Allium cepa* L., *Brassica oleracea* L., *Solanum lycopersicum* L., and *Lactuca sativa* L. [89]. 

Flavonols are recognized for their beneficial properties, including antioxidant, anti-inflammatory, antibacterial, and antiviral effects. Their antioxidant capacity plays a crucial role in neutralizing free radicals and reducing oxidative stress, which is commonly associated with aging and various chronic diseases such as cardiovascular disease, cancer, and neurodegenerative disorders [11,88]. For example, quercetin, the most prevalent flavonol, has demonstrated therapeutic efficacy against hepatic and renal toxicity, as well as metabolic, cardiovascular, and cerebrovascular diseases. Its neuroprotective properties, particularly in the context of Alzheimer’s disease, arise from its ability to inhibit tau phosphorylation, reduce beta-amyloid aggregation, and prevent oxidative damage [90]. Additionally, the flavonol rutin offers protection against conditions such as gout by lowering uric acid levels, reducing inflammation, and inhibiting xanthine oxidase activity [91]. Rutin also decreases pro-inflammatory cytokines and enhances the activity of antioxidant enzymes, thereby contributing to its extensive anti-inflammatory and neuroprotective properties [92,93]. Another significant flavonol, isorhamnetin, found in plants such as *Hippophae rhamnoides* L., *Ginkgo biloba* L., *Vaccinium corymbosum* L., and *Vaccinium myrtillus* L. has exhibited cardioprotective effects through the attenuation of oxidative stress and inflammation. This is achieved by obstructing key inflammatory signaling pathways, including JNK and AKT/IKK [94]. 

Research has demonstrated that morin serves a protective function against ischemic lesions and aids in the recovery from cerebral reperfusion injuries in animal models, thereby underscoring its significant role in supporting cerebrovascular health [95,96]. The primary sources of flavonols along with their biological activities are provided in Table 2. 

##### Flavanones 

Flavanones, also referred to as dihydro-flavones, are colorless compounds that exhibit slight solubility in water. However, upon undergoing alkalization, they become water-soluble phenolates, which are characterized by a yellow coloration [11]. Eriodyctiol, hesperetin, and naringenin [89], present in the epicarp and mesocarp of various citrus fruits, are primarily linked to the bitter taste associated with these fruits. The health benefits attributed to flavanones include antioxidant, anti-inflammatory, lipid-lowering capacities, cholesterol reduction, and combating atherosclerosis [11]. Naringerin is the primary flavonoid found in pomelo peel [119] and has been shown to reduce cardiovascular and liver toxicity, induced by 5-fluorouracil in mice with colorectal cancer [120]. Other studies involving ovariectomized rats indicate that naringerin can help combat obesity, insulin resistance, and the enlargement of fat cells (adipocyte hypertrophy) that occur because of estrogen deficiency [121]. Eriodictyol may also have anticancer properties. Research by Li W. et al. demonstrated that it can induce apoptosis in glioma cells by blocking the PI3K/Akt/NF-κB signaling pathway [122]. Other studies have shown that eriodictyol can attenuate intestinal tissue damage caused by 2,4,6-trinitrobenzene sulfonic acid [123] in rats and may prevent diabetic nephropathy induced by streptozotocin through its hypolipidemic, antioxidant, and anti-inflammatory effects [124]. Hesperetin, which can be extracted from citrus fruits such as *Citrus sinensis* (L.) Osbeck and *Citrus limon* (L.) Osbeck, has also been studied for its potential in combating breast cancer. Research by Li et al. found that hesperetin inhibits the metastasis of human triple-negative breast cancer cells (MDA-MB-231) that is induced by transforming growth factor beta 1 [125]. Alotaibi, K.S. et al. evaluated the antioxidant effect of hesperetin following the exposure of rats to hepatotoxicity induced by bisphenol A. Their results showed that hesperetin reduced the levels of pro-inflammatory cytokines and led to the overexpression of the nuclear transcription factor erythroid 2 in the liver [126]. The main sources of flavanones and their biological activities are presented in Table 3. 

##### Flavan-3-ols/proanthocyanidins

Flavan-3-ols also known as catechins, are a type of flavonoid compound found in plants. Unlike other flavonoids, they can exist as monomers oligomers, polymeric aglycones, or esters with gallic acid, forming compounds such as gallocatechin, epicatechin, and epigallocatechin [38]. The most widespread proanthocyanidins in plants are detailed in Table 4 [155]. Flavan-3-ols are abundantly present in a variety of sources, including woody and herbaceous plants, cocoa, tea, fruits (such as bananas, apples, grapes, blueberries, peaches, and pears), jams, lentils, beans, seeds, and beverages like wine, cider, fruit juices, and beer [11,156]. Research shows that epicatechin enhances the activity of the body’s antioxidant enzymes (glutathione peroxidase, superoxide dismutase, catalase) while also increasing glutathione levels. It has been found to reduce levels of malondialdehyde, nitric oxide, interleukin-1β, and tumor necrosis factor-alpha [157,158]. Additionally, epicatechin induces apoptosis and antagonizes proapoptotic androgenic actions in breast and prostate cancer cells through the membrane androgen receptor ZIP9 [159]. Gallocatechin exhibits a neuroprotective effect by reducing intracellular free radicals and inhibiting the phosphorylation of extracellular signal-regulating kinase and C-Jun N-terminal kinase [160]. Silver nanoparticles derived from gallocatechin and chitosan have been shown to promote wound healing in diabetic rats [161]. Epigallocatechin has exhibited antiviral effects in vitro by disrupting the DNA replication cycles of viruses, such as the hepatitis B virus, herpes simplex virus, and adenovirus. It also possesses antifungal properties against *Candida albicans* and antibacterial effects against *Staphylococcus aureus* and *Stenotrophomonas maltophilia* [162].

##### Anthocyanins 

Anthocyanins are the pigments responsible for the various colors including pink, red, purple, violet, blue, and bluish-black, found in the epidermis [89] of plants, flowers, fruits, seed skins, and sometimes leaves [8]. Table 5 lists the most common anthocyanidins found in foods [175] such as cranberries, blackcurrants, raspberries, strawberries, blueberries, and blackberries with levels ranging from 100 to 700 mg/100 g fresh product [11,175]. The highest concentrations of anthocyanins are identified in elderberries and aronia, with levels between 1.4 and 1.8 g/100 g of fresh product [175,176]. 

The color and stability of anthocyanins are influenced by factors such as pH, light, temperature [175], and chemical structure [11]. The health benefits of anthocyanins include their potential to combat cardiovascular diseases (such as hypertension, dyslipidemia, atherosclerosis, and oxidative stress), neurodegenerative diseases (including Parkinson’s and Alzheimer’s), various cancers (breast, colon, prostate, and lung), and diabetes mellitus [177]. Cyanidin has been noted for its role in combating obesity by inhibiting the differentiation of preadipocyte cells [178]. It stimulates calcium release from the endoplasmic reticulum, raises intracellular calcium levels in preadipocyte cells, activates phospholipase C, and promotes the production of inositol 1,4,5-triphosphate, which aids in calcium mobilization. In studies involving gastric cancer cells, such as the MKN-45 line, cyanidin induces apoptosis, inhibits cell proliferation, and prevents cell motility and migration [179]. Delphinidin prevents the spread of both gastric and intestinal cancer in vitro [179], effects that have been observed in human epithelial colorectal adenocarcinoma cells. Additionally, its anti-inflammatory properties have been validated in vitro in conditions such as psoriasis and psoriatic arthritis [180]. Rosinidine has been found to lower blood sugar levels, decrease insulin levels, and potentially improve pancreatic function in rats with type 2 diabetes induced by streptozotocin. Additionally, it mitigates rotenone-induced neurotoxicity in rats by reducing neuroinflammation, lowering neurotransmitter levels, and enhancing locomotor function [181,182]. Pelargonidin is associated with a reduced risk of Alzheimer’s disease and helps decrease obesity by inhibiting glucose consumption, triglyceride accumulation, and adipogenesis in a murine fibroblast cell line [183]. It also exhibits a chemoprotective effect against colorectal cancer [184]. 

Research has demonstrated the anticancer effects of malvidin in vivo, revealing a reduction in the migration and proliferation rates of metastases, as well as the induction of apoptosis and enhanced levels of autophagy in cancer cells [185]. The antiulcer effects observed in vivo were indicated by decreased myeloperoxidase levels and elevated levels of catalase, superoxide dismutase, and reduced glutathione [186].

**Table 5:** Natural sources of anthocyanidins and their bioactivity.

Name of the Anthocyanidin	Bioactivities	Natural Sources	Ref.
Cyanidin	Cardioprotective, neuroprotective, antioxidant, antidiabetic, anti-inflammatory, anticancer	Malus *pumila* Kitam., *Vaccinium myrtillus* L., *Aronia melanocarpa* (Michx.) Elliott, *Vaccinium oxycoccos* L., *Amelanchier arborea* (F. Michx.) Fernald, *Pyrus communis* L., *Empetrum nigrum* L., *Sambucus nigra* L., *Rubus fruticosus* L., *Ribes nigrum* L., *Prunus armeniaca* L., *Morus alba* L., *Punica granatum* L., *Daucus carota* L., *Solanum melongena* L.	[11,187,188,189,190]
Delphinidin	Anti-inflammatory, anticancer	*Clitoria ternatea L*., *Aristotelia chilensis* (Molina) Stuntz, *Hibiscus sabdariffa* L., *Vaccinium myrtillus* L., *Vitis vinifera* var. *tinctorialis* Risso, *Ribes nigrum* L., *Secale cereale* L., *Vaccinium myrtillus* L., *Morus alba* L., *Punica granatum* L., *Solanum melongena* L., *Pisum sativum* L., *Capsicum annuum* L., *Hippophae rhamnoides* L., *Nelumbo nucifera* Gaertn	[11,188,189,191,192,193,194]
Rosinidine	Antidiabetic, neuroprotective	*Catharanthus roseus* (L.) G.Don, *Primula rosea* Royle	[195,196]
Pelargonidin	Neuroprotective, anticancer, chemoprotective	*Sambucus nigra* L., *Punica granatum* L., *Phaseolus vulgaris* L., *Daucus carota* L., *Solanum tuberosum* L., *Raphanus sativus* L.	[11,183,189,197,198]
Malvidin	Anticancer, antiulcer	*Vaccinium myrtillus* L., *Vitis vinifera* var. *tinctorialis* Risso, *Solanum tuberosum* L., *Nelumbo nucifera* Gaertn	[11,189,199,200]
Peonidin	Neuroprotective	*Vaccinium myrtillus* L., *Vaccinium macrocarpon* Aiton, *Vaccinium oxycoccos* L., *Daucus carota* L., *Solanum tuberosum* L.	[11,189,201]
Petunidin	Antiosteoporosis	*Vaccinium myrtillus* L, *Vaccinium macrocarpon* Aiton, *Vitis vinifera* var. *tinctorialis* Risso, *Secale cereale* L, *Solanum melongena* L, *Solanum tuberosum* L.	[11,189,202,203]

##### Chalcones

Most of these compounds are polyhydroxyl aromatic compounds [204] that are yellow in color and possess either lipophilic or hydrophilic characteristics depending on the structural features [11]. Chalcones, as presented in Table 6, along with their structural analogs, exhibit various bioactivities, including anticancer, antidiabetic, anti-inflammatory, antimicrobial, antiparasitic, and antioxidant properties [40,205]. Phloretin is one of the most common chalcones found in food [40]. This compound exhibits antifungal effects against *Candida albicans*, inhibiting biofilm formation, the transmission from yeast to hyphae, and the secretion of protease and phospholipase [206]. Additionally, phloretin may help combat diabetic nephropathy [207], multiple sclerosis, and nonalcoholic fatty liver disease [208,209]. Butein has been shown to have antiulcer effects, which are indicated by an increase in the level of prostaglandin E2 and a decrease in the levels of messenger ribonucleic acid, cyclooxygenase-1, and cyclooxygenase-2 in the stomach. Moreover, it may exert antitumor effects against nasopharyngeal carcinoma and p53 mutant cancer cells [210,211]. Isoliciritigenin has demonstrated antitumor activity in various types of cancer, including breast, colon, gastrointestinal, lung, ovarian, leukemia, and melanoma. It induces ferroptosis in gallbladder cancer by reducing glutathione peroxidase 4 levels and activating heme oxygenase 1 [212,213,214]. Furthermore, it exhibits anti-inflammatory effects by inhibiting nuclear factor kappa B, NOD-like receptor family pyrin domain containing 3, and mitogen-activated protein kinase, while activating nuclear factor erythroid 2 [215].

### 3.3. Quercetin—The Most Important Flavonol Compound from the Flavonoid Group

The term ”quercetin” is derived from the Latin word “*quercetum*”, which refers to *Quercus robur* commonly known as oak [233]. The first chemical synthesis of quercetin was accomplished in 1904, by Kostanecki et al., a distinguished Polish chemist [234], utilizing 5,7,3’,4’-tetramethyl eriodictyol chalcones as the starting material [235]. In 1930, quercetin in the form of quercetin 3-O-rutinoside was synthesized from oranges [236]. Quercetin is recognized as a significant antioxidant and is employed as a nutraceutical component in various pharmaceutical, cosmetic, and food formulations due to its protective effects on cells against oxidative stress and damage [237]. Furthermore, it is used as an adjuvant in the management of multiple health conditions owing to its anti-obesity, anticarcinogenic, antiviral, antibacterial, and anti-inflammatory properties [238].

Studies have shown that it can be used in cancer treatment. Quercetin inhibits the proliferation of various cancer cells, including those from the colon, prostate, liver, pancreas, and lungs. This effect is attributed to its antioxidant properties [238]. Additionally, quercetin has been used in various randomized trials, as an adjuvant in doses of 1500 mg/day and 1000 mg/day, to treat SARS-CoV-2 infection [239]. Patients receiving quercetin alongside specific antiviral medications, such as remdesivir and favipiravir, showed decreased serum levels of C-reactive protein, lactate dehydrogenase, and alkaline phosphatase. Their length of hospitalization was also reduced [240]. Quercetin can combat inflammatory diseases of the central nervous system. It has been shown to inhibit the expression of lipocalin-2 in macrophages and microglial cells. Additionally, it decreases the production of nitric oxide and the expression of pro-inflammatory factors, including inducible nitric oxide synthase and cyclooxygenase-2 (COX-2). It also reduces the expression of M1 markers, such as interleukin-6, tumor necrosis factor (TNF)-α, and interleukin-1β, as well as the expression of chemokines [241]. In preclinical studies, quercetin was observed to inhibit ferroptosis associated with type 2 diabetes mellitus by manifesting protective effects on pancreatic β cells, leading to increased insulin secretion [242]. Due to the multitude of beneficial effects that this compound presents, it is currently only used as a dietary supplement in powder or capsule form [243].

#### 3.3.1. Natural Sources, Chemical Structure, and Physico-Chemical Properties of Quercetin

Quercetin-like flavonoids (quercetin glycosides) are widely distributed in various species of plants, fruits, and vegetables [243,244]. *Malus domestica* (Suckow) Borkh., *Pyrus communis* L., *Solanum lycopersicum* L., *Ipomoea batatas* (L.) Lam., *Sambucus canadensis* L. *Fragaria ananassa* Duchesne, *Capsicum annuum* L., *Carica papaya* L., *Lactuca capitata* DC., *Phaseolus vulgaris* L., *Vitis vinifera* L., *Prunus dulcis* (Mill.) D.A. Webb, *Pistacia vera* L., *Juglans regia* L., and *Coffea arabica* L. are the most well-known sources of quercetin [245,246].

The amount of quercetin in plants is most often identified by HPLC (High-Performance Liquid Chromatography), according to Table 7 [245], and may differ in plants depending on the variety, cultivation conditions, and mode of exposure to light [247]. 

Quercetin is a yellow-colored plant pigment [233] found in plants, vegetables, and fruits in glycosidic form (i.e., bound to one, two, or more saccharide molecules). Glycosylation of quercetin occurs most often at the OH free group at position 3, but less often it may occur at 3’, 4’, or 7-position [269]. The most common quercetin glycosides in plants are shown in Table 8.

Rutoside, also known in the literature as rutin [11,282,283], isoquercitrin, and quercitrin are the most commonly occurring glycosidic forms of quercetin in nature [284]. It is considered that the saccharide group may change the solubility, absorption, and in vivo action of the quercetin molecule because, according to studies, aglyconic quercetin has low solubility in water. After all, it is a lipophilic compound, and in humans, it has low oral bioavailability [276,285]. In dietary supplements, quercetin is used in the aglyconic form in daily doses ranging from 100 to 500 mg [286]. 

Quercetin is only found in plants, fruits, and vegetables because animals are unable to synthesize the flavonoid nucleus [287]. It is a derivative of phenylalanine, generally produced by the phenylpropanoid route [288].

The flavonoid compounds, including quercetin, are synthesized in plants via the phenylpropanoid pathway. L-phenylalanine is the initial precursor of this pathway, from which quercetin is derived. This amino acid is produced through the shikimic acid pathway, which, after a series of enzymatic reactions, is converted into chorismate and then into L-phenylalanine [289].

**Figure 5:**
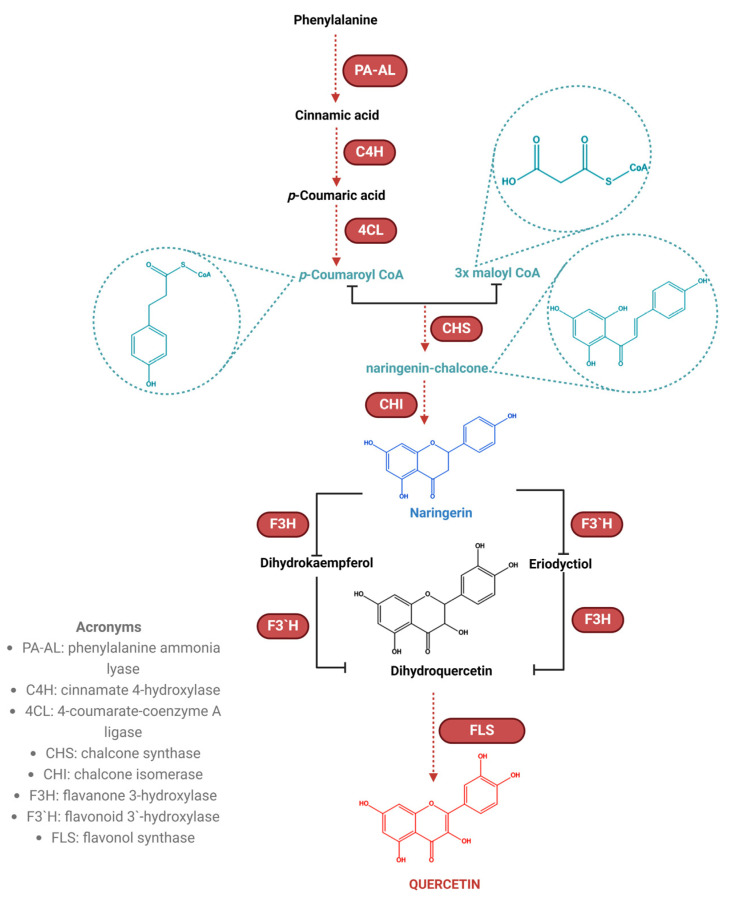
Biosynthesis of quercetin in plants (scheme made with the Biorender program).

In the context of quercetin biosynthesis (as illustrated in Figure 5), L-phenylalanine undergoes dehydrogenation catalyzed by phenylalanine ammonia-lyase, resulting in the formation of cinnamic acid. Subsequently, cinnamic acid is hydroxylated at position 4 via the action of cinnamate-para-hydroxylase, leading to the production of p-coumaric acid. p-Coumaric acid is then converted to p-coumaroyl-CoA through the transformation of its carboxyl group mediated by 4-coumarate-Coenzyme A ligase [233,290]. 

Then, by condensation with three molecules of malonyl-CoA, under the influence of calcon synthetase, this compound is transformed into chalcon–naringerin. At this time, part of the rings of the flavonoid molecule has been obtained, namely, rings A and B. Calcon–naringerin, under the influence of calcon isomerase, through the isomerization reaction, is transformed into naringerine flavanone. By hydroxylating naringerin at the C_3_ and C_3’_ positions, dihydrokaempferol is formed under the action of flavanon-3-hydroxylase and eriodicthiol is formed under the influence of flavonoid-3’-hydroxylase. Then, dihydroquercetin results, by hydroxylation reactions, in the positions C_3’_ of dihydrokaempferol under the influence of flavonoid-3’-hydroxylase and C_3_ of eriodyctiol under the influence of flavanon-3-hydroxylase. Further, dihydroquercetin [291] is converted under the influence of flavonol synthetase into quercetin, due to the formation of the double bond between C2 and C3 in the pyranic nucleus [283]. Flavanon 3-hydroxylase belongs to the family of enzymes known as dioxygenases. These enzymes use 2-oxoglutarate to catalyze the reactions of adding oxygen to their substrates [292]. Enzymes that are involved in quercetin biosynthesis are part of the cytochrome-P450 family of enzymes and can be found in the cytoplasmic membrane, mitochondria, or in the membrane of various cytoplasmic organelles such as plastids (chloroplasts), nuclei, and plant vacuoles [233]. 

Studies show that traditional sources of quercetin are increasingly low and are no longer sufficient to meet current demands [293]. Thus, extracting it from native plants or chemical synthesis in bulk is becoming increasingly difficult [289]. Currently, quercetin is synthesized by various synthetic methods of metabolic and genetic engineering, using microorganisms as hosts [293]. This synthesis differs significantly from the biosynthesis produced in plants, which uses specific metabolic pathways for the synthesis of flavonoids. Although microorganisms can produce precursors necessary for the synthesis of aromatic amino acids (phenylalanine), they cannot convert these precursors into polyphenolic compounds via the phenylpropanoid pathway [292]. The most widely used microorganisms for the production of polyphenols, using metabolic engineering, are the bacterium *Escherichia coli* and the yeast *Saccharomyces cerevisiae*. *Saccharomyces cerevisiae* is a eukaryotic organism, which can undergo post-translational changes (glycosylation) and possesses intracellular compartments similar to plants [294]. *Escherichia coli* is generally used in the production of quercetin derivatives: quercetin *3-O*-(6-deoxytalose) [295], 3-O-xylosyl-quercetin [296], quercetin 3-O-rhamnoside, and quercetin 3-O-galactoside [297], etc.

By genetic engineering, Marin L. et al. synthesized quercetin in actinomycete organisms (*Streptomyces coelicolor* and *Streptomyces albus*) using heterologous expression of their plant biosynthetic gene pathways. For the biosynthesis of quercetin, it was necessary to activate the flavonoid enzyme 3’-hydroxylase. This enzyme favors the introduction of a hydroxyl group on the B nucleus of kaempferol, at the 3’ position. After kaempferol was produced by the host microorganism, the gene encoding the flavonoid enzyme 3’-hydroxylase was cloned into a plasmid (pQR). The new plasmid was transformed into protoplasts of the species *Streptomyces albus* and *Streptomyces coelicolor*, which allows the expression of the enzyme. Cultures from recombinant strains that were confirmed as positive were analyzed, by HPLC-MS chromatography, to identify and quantify the quercetin produced [298].

The name of quercetin according to IUPAC (International Union of Pure and Applied Chemistry) is 3,3’,4’,5,7-pentahydroxyflavone or 3,3′,4′,5,7-pentahydroxy-2-phenylchromen-4-one [245], and has the molecular formula (C_15_H_10_O_7_) [238]. It is also known as sophoretin [299] meletin, xanthaurin, quercetol, or quertine [300]. According to the chemical structure presented in Table 8, quercetin has the basic skeleton C_6_-C_3_-C_6_, specific to flavonoids, with the difference that in positions 3, 5, 7, 3’, and 4’, it has five hydroxyl groups, in position 4, it has the oxo group, and between C_2_-C_3_, there is a double bond [301]. The presence of the free hydroxyl groups of positions 3, 5, and 7, the resorcinolic ring A, the catecholic ring B, and the double bonds of the oxo group provide quercetin with particularly strong antioxidant properties when it reacts with free radicals [269,301,302].

According to the physicochemical properties, quercetin (Table 9) has a bitter taste; it is a yellow powder, very little soluble in water, with a high molecular weight of 302.24 g/mol [238,284,303,304]. The presence of the five hydroxyl groups in the structure makes the quercetin molecule lipophilic. The glycosidic derivatives of quercetin, depending on the nature of the substituents in the structure, can be lipophilic or hydrophilic. The structure of quercetin can be broken down by heating to high temperatures, and when burned, it emits sour smoke and irritating vapors [284].

#### 3.3.2. Metabolism, Bioavailability, and Delivery Systems Based on Quercetin

The metabolism of quercetin consists of the transformation of glycoside quercetin into aglycone quercetin after ingestion, chewing, digestion, and absorption under the action of the oral or intestinal flora and the action of β-glycosidases. Aglycone quercetin after hydrolyzing, can easily cross intestinal membranes through the process of passive diffusion, due to lipophilia with the help of the intestinal co-transporter sodium/glucose-1 [306]. After intestinal absorption, quercetin is metabolized into enterocytes, transported to the liver through the portal vein, and then binds strongly to plasma albumin [306]. The maximum level of quercetin in plasma is reached after 0.7–7.0 h [307]. Metabolism products resulting in the small intestine and liver (glucuronidated, methylates, sulfates, 3’-O-monomethyl-quercetin, and 4’-O-monomethyl-quercetin) from the second phase of enzymatic metabolism will be further distributed in the tissues of the body (liver, lungs, kidneys, heart, small intestine), and after the manifestation of the effect, they will be eliminated through the renal, fecal, and respiratory systems [308,309]. The period of residence of quercetin in the body can vary between 20 and 72 h [310]. 3-hydroxyphenyl acetic acid, hippuric acid, and benzoic acid [311] are considered to be metabolizing byproducts, metabolized in the liver and eliminated from the body via the biliary tract [312] or pulmonary [313]. Quercetin exhibits prolonged elimination from the body, a half-life that can range from 11 to 28 h, and an average terminal half-life of about 3.5 h [301]. Unabsorbed quercetin is degraded by the colonic microbiota, to phenolic acids that are absorbed, and then transported to the liver for further conjugation [306].

Studies show that quercetin has poor bioavailability after oral administration [308]. Following administration, it may suffer the effect of the first intestinal passage [307]. Phase II metabolism significantly affects the bioavailability of quercetin in humans, due to the fact that much of it is excessively metabolized in the liver, before reaching the systemic circulatory system [308]. Normally, after oral administration, the concentration of quercetin in human plasma is very low, in the nanomolar domain, as is the half-life of the metabolites. The elimination time of quercetin is approximately 25 h and may be delayed if co-administered with fat-containing foods [307]. Plasma concentration and half-life are considered to be increased only if the quercetin dose is increased or given repeatedly [35].

According to studies, the low bioavailability of quercetin is due to poor water solubility due to increased lipophilicity, decreased absorption in the gastrointestinal tract, instability in the stomach and intestine, short half-life, low permeability, oxidative degradation, and high effect of the first hepatic passage [314,315,316,317,318]. These factors, responsible for the low oral bioavailability of quercetin, prevent its use as a pharmaceutical agent in the medical field. To avoid these factors, in recent years, a lot of research has been performed on the development of new delivery systems that improve its bioavailability and solubility, but at the same time increase its biological activities. 

Quercetin bioavailability can be improved by including it in various delivery systems such as nanosuspensions, nanoemulsions, self-emulsifying/microemulsifying delivery systems, cocrystals, niosomes, lipogels, liposomes, etc., or by complexing with cyclodextrin [238,319,320,321,322].

##### Nanosuspensions and Nanoemulsions

Li H. et al. showed in their study that the oral absorption of quercetin can be significantly improved if it is included in nanosuspensions with phase II metabolic inhibitors, such as piperine and sodium oleate [323]. Quercetin nanosuspensions obtained using the liquid antisolvent precipitation method showed particle sizes of 202.15 ± 2.45% nm, a zeta potential of −20.26 ± 1.32 mV, and an entrapment efficiency of 95.22 ± 2.45%. Studies conducted on Caco 2 cells showed that cell viability was over 90%, suggesting that the use of quercetin nanosuspension may be safe [324]. 

Quercetin-loaded nanoemulsions obtained by the ultrasonication technique are another way to increase this oral bioavailability. According to an in vivo study conducted on rats, quercetin nanoemulsions produced by Mahadev M. et al. showed a protective and therapeutic effect against streptozotocin-induced diabetes mellitus after 21 days of treatment. They also controlled body weight and blood glucose levels and inhibited high serum lipid levels, tissue damage, and oxidative stress markers [325]. By complexing and self-assembling quercetin with Tween 80, Captex 355, sodium alginate, and lecithin from soybean, O/W nanoemulsions can be obtained. The results of the stability study showed that the nanoemulsions were stable in the pH range of 6.5–9 during storage at a temperature of 37 °C. The entrapment efficiency was between 56 and 92%, and animal experiments showed that these nanosuspensions can regulate serum and liver cholesterol levels [326]. The benefit of encapsulating quercetin in O/W nanoemulsions is given by their resistance to gravitational separation, aggregation (flocculation or coalescence), and digestion from the gastrointestinal tract, which increases their bioavailability [327].

##### Self-Emulsifying/Microemulsifying Delivery Systems

The development of self-emulsifying super saturable quercetin delivery systems represents a new strategy to increase its bioavailability. According to Arvind Sirvi et al., by oversaturation, an increased concentration of quercetin in the gastrointestinal lumen can be ensured if HPMC (hypromellose acetate succinate) is introduced into the formulation as an inhibitor of its precipitation in crystalline form [328]. Jaisamut P. et al., by self-microemulsifying quercetin with Capryol 90, Cremophor EL, and Labrasol, increased the oral bioavailability of quercetin. This was observed following an in vivo pharmacokinetics study in rats where the area under the formulation curve containing quercetin and resveratrol increased approximately ninefold for quercetin and three times for resveratrol, compared with non-formulated compounds. The in vitro cell study showed an antioxidant effect against gastric AGS and intestinal Caco-2 cells and a cytoprotective effect against HT-29 colorectal cancer cells [329].

##### Cocrystals 

Using the solvent evaporation technique, Wu N. et al. prepared quercetin cocrystals with different stoichiometries, using nicotinamide as a cocrystal former. Pharmacokinetic studies from in vivo experiments suggested that oral absorption of quercetin could increase by approximately 4-fold if it is included in nicotinamide cocrystals [330]. Smith A.J. succeeded by cocrystallization to increase the solubility of quercetin–caffeine cocrystal in ethanol/water solvent mixture by 14-fold, compared to quercetin dihydrate [331].

##### Niosomes and Liposomes

Niosomes are composed of nonionic surfactants that self-assemble in aqueous media to form a bilayer structure. These drug carriers are biocompatible, non-immunogenic, and biodegradable. They can be used to encapsulate compounds with low solubility or molecules that can be easily degraded due to their amphiphilic nature [332]. Murugesan N. et al., increased the bioavailability of quercetin by including it in vector systems, such as niosomes, prepared by the thin film hydration method, using cholesterol and Span 60 [333]. 

The inclusion of quercetin, which is a hydrophobic compound, in lipogels through the absorption process may represent another strategy to increase its bioavailability. Quercetin liposomes were obtained by the thin film rehydration technique, using phosphatidylcholine, after which they were allowed to be absorbed into the semi-interpenetrating polymer network of chitosan and gelatin hydrogel [334]. Caddeo C. et al. included quercetin in Eudragit-coated liposomes to protect it from the gastric environment and allow it to be delivered to the gut [335]. Compared to free quercetin, liposomal quercetin was found to be much more effective in reducing ROS production, modulating insulin resistance [336], penetrating the blood–brain barrier to manifest neuroprotective effects [337], manifesting antitumor effects against colon cancer cells [338], and the induction of apoptosis and the reduction epidermal growth receptor factor expression in colorectal cancer cells [339]. 

##### Inclusion Complexes with β-Cyclodextrins

Another way to increase the solubility and stability of quercetin in water would be to complexify quercetin with β-cyclodextrins [340] to form advantageous inclusion complexes [341]. The use of hydroxypropyl-β-cyclodextrin to prepare quercetin inclusion complexes is advantageous as it increases the water solubility of quercetin by 630-fold [342]. Wangsawangrung N. et al. encapsulated quercetin in hydroxypropyl-β-cyclodextrin and then included this content in a polyvinyl alcohol-based hydrogel, through the freeze–thaw process. The formulation enhanced the water solubility and stability of quercetin and exhibited an entrapment efficiency of 90.50 ± 1.84% and a loading capacity of 4.67 ± 0.13% [343]. Papakyriakopoulou P. et al. prepared quercetin and methyl/hydroxypropyl-β-cyclodextrin nasal powders, and then, the inclusion complex was introduced into mannitol/lecithin microparticles by the spray drying method in order to increase the bioavailability of quercetin in the brain. The results of the study showed that in the pH 7.4 environment, at a temperature of 37 °C, the solubility of quercetin in water increased by 19–35 times, and the permeability increased by about 17 and 48 times, compared to that of pure quercetin, and the in vitro release using Franz cells and powder transportation through the nasal mucosa of the rabbit were much faster compared to pure quercetin [344]. Other ways to increase the bioavailability of quercetin are presented in Table 10.

#### 3.3.3. Health Benefits of Quercetin

Although the most important action of quercetin is the antioxidant action given by the capture and inactivation of reactive oxygen species (ORS), inhibition of lipid peroxidation, and capture of prooxidant transitional metal ions [11,320], quercetin is also known to produce other effects: antihypertensive, antiatherogenic, neuroprotective, anticancer, antitumor, antiulcer, antiallergic, antiviral, and antibacterial [282,285,362].

##### Antioxidant and Anti-Inflammatory Effects

The antioxidant effect of quercetin is due to its ability to neutralize free radicals and prevent oxidative stress. By neutralizing free radicals, it acts as an electron donor, reducing reactive oxygen species (ROS), e.g., hydroxyl radical (OH-), hydrogen peroxide (H_2_O_2_), superoxide (O_2_^−^), and free radicals, e.g., nitric oxide (NO) and DPPH [327]. Lesjak M. et al. observed through FRAP (Ferric Reducing Antioxidant Power) and DPPH (2,2-diphenyl-1-picrylhydrazil) studies, that aglyconic quercetin has the greatest antioxidant effect, and in the case of its derivatives, the antioxidant effects increase directly proportional to the number of free hydroxyl groups present in the structure [363]. Giordano, M.E. and Lionetto, M.G showed, in a study conducted on HeLa cells loaded with ROS-sensitive CM-H, that quercetin had significantly detectable antioxidant capacity at 5 μM and peaked at 25 μM relative to hydrogen peroxide. The antioxidant effect of quercetin could be due to the known anti-radical activity of the catechol group (3′,4’-dihydroxy) of the B ring and the double bond between the carbon 2 and carbon 3 of the C ring conjugated to the keto group at position 4 [364]. Suematsu, N. et al. investigated the neuroprotective effects of quercetin against H_2_O_2_-induced apoptosis in human SH-SY5Y neuronal cells. The results showed that both H_2_O_2_-mediated cytotoxicity and lactate dehydrogenase release were suppressed by quercetin. In addition, quercetin increased the expression of the Bcl-2 gene and inhibited the activation of caspase, which leads to DNA fragmentation, the appearance of apoptosis, and subsequent cell death [365]. 

Studies show that oxidative stress can activate several transcription factors that can influence the genes involved in inflammatory pathways differently. Thus, the most important transcription factor is nuclear factor kappa B (NF-κB), which is normally bound to IκB proteins in the cytoplasm and thus is in an inactive form. When ROS enter cells through membrane receptors, they can degrade the IκB protein by phosphorylation, following the activation of NF-κB kinase (IKK). Once degraded, NF-κB is activated and then translocated to the nucleus, where it binds to the DNA consensus sequence of various target genes, which are involved in the inflammatory process (tumor necrosis factor (TNF)-α, interleukin (IL)-1β and IL-6) [366]. Due to its ability to reduce oxidative stress, quercetin may protect cells from lipopolysaccharide-induced lung damage, according to lung epithelial cell studies. Quercetin can also almost completely reduce the levels of mRNA and NOX2 (ROS-generating enzyme) in lung epithelial cells. It can suppress the nuclear translocation of NF-κB and inhibit the expression of pro-inflammatory cytokines such as TNF-α and IL-1β [366]. In another study conducted on glial cells, quercetin along with resveratrol was observed to decrease lipopolysaccharide-induced mRNA levels of two pro-inflammatory genes, IL1-α and TNF-α. This may suggest that resveratrol and quercetin protected dopaminergic neuronal cells from inflammation-mediated apoptotic death [367]. Another study showed that quercetin can exhibit an anti-inflammatory effect in vivo, which was due to the inhibition of cyclooxygenase-1 (COX-1) and lipoxygenase-12 (LOX-12) enzymes, both of which are involved in inflammatory processes. COX-1 is the enzyme involved in the conversion of arachidonic acid to prostaglandins, which are mediators of inflammation. By inhibiting COX-1, quercetin reduces the production of pro-inflammatory prostaglandins, thereby reducing inflammation. LOX-12 is the enzyme involved in the synthesis of leukotrienes, which are other pro-inflammatory substances. By inhibiting this enzyme, quercetin contributes to lowering leukotriene levels, thus having an anti-inflammatory effect. By inhibiting these enzymes, quercetin can thus reduce the activation of macrophages and neutrophils [363].

The biosynthesis of prostaglandins and nitric oxide is involved in inflammation, and the inducible isoforms of nitric oxide synthase (iNOS) and cyclooxygenase (COX-2) are responsible for producing large amounts of these pro-inflammatory mediators. C-reactive protein (CRP) is an acute-phase protein synthesized by hepatocytes, and its elevated serum levels are seen as a sign of chronic inflammation. According to studies on cells derived from human Chang Liver hepatocytes (CHL), quercetin can decrease the production of inflammatory molecules COX-2, nuclear factor-kappa B (NF-κB), activator protein 1 (AP-1), mitogen-activated protein kinase (MAPK), nitric oxide reactive synthase (NOS), and C-reactive protein (CRP) [368]. In experimental models on mice sensitized to ovalbumin, it was observed that quercetin can be used for the clinical treatment and prevention of allergic diseases, such as allergic rhinitis and allergic conjunctivitis through several mechanisms of action. It modulated the immune response by balancing the activity of T helper 1 and T helper 2 cells. If there is an imbalance between these cells, then an excessive amount of IgE antibodies is secreted in the body, which are involved in triggering allergic reactions. Another observation was that it inhibited the activity of inflammatory mediators: eosinophils, neutrophils, and macrophages, thus being able to alleviate the two allergic symptoms. By inhibiting the NF-κB signaling pathway, quercetin reduced the expression of inflammatory mediators such as COX-2, p-IKBα, and nuclear-p65, thus contributing to the reduction of inflammation [369].

##### Neuroprotective Effect 

**Figure 6:**
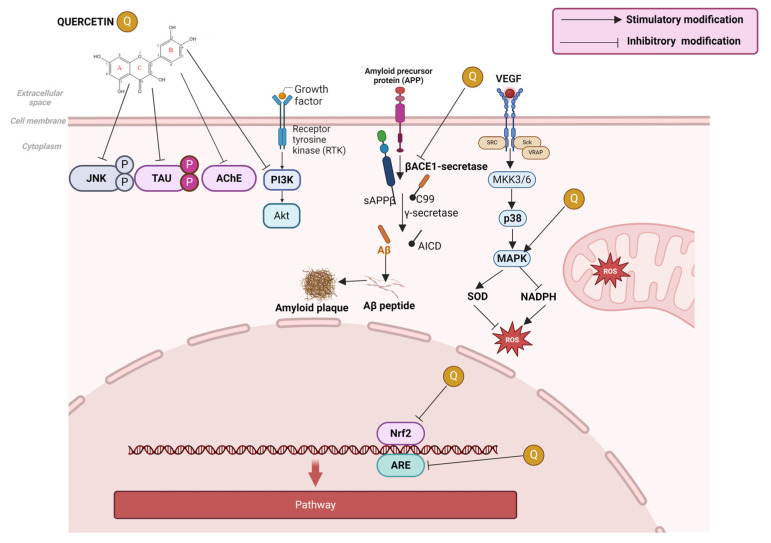
Schematic representation of quercetin’s role in Alzheimer’s disease pathogenesis (scheme prepared with the Biorender program). Legend: Q—quercetin, JNK—c-Jun N-terminal Kinases, TAU—TAU protein, AChE—acetylcholinesterase, PI3K—Phosphoinositide 3 Kinase, Akt—protein kinase B, ΒACE1-secretase—β-Secretase 1, Aβ—β amyloid, VEGF—Vascular Endothelial Growth Factor, MKK3/6—mitogen-activated protein kinases 3 and 6, p38—protein kinase 38, MAPK—mitogen-activated protein kinases, SOD—superoxide dismutase, NADPH—Nicotinamide Adenine Dinucleotide Phosphate, ROS—reactive oxygen species, Nrf2—nuclear factor erythroid 2, ARE—Antioxidant Response Element.

In a study conducted on rats, the neuroprotective effect of quercetin, in combination with piperine, in the symptoms of Parkinson’s disease, induced by rotenone and iron supplementation, was highlighted. The study showed that quercetin prevents dopaminergic neuronal degeneration induced by rotenone and iron supplementation, either by controlling ROS production, by producing anti-inflammatory mechanisms, or by mitochondrial energy restoration mechanisms. The antioxidant effect of quercetin on neuronal cells was observed as a result of increasing the level of GSH, decreasing the level of malondialdehyde and nitrates, and through the ability to chelate Fe ions [370]. A series of studies claim that quercetin, through its ability to neutralize and eliminate ROS, can prevent damage to lipids in neuronal cells, stimulate the enzymatic systems (superoxide dismutase and catalase) of defense, and inhibit the activity of microglial cells [314,371]. According to Zaplatic E. et al., they showed that inactivating nuclear factor erythroid 2 (Nfr2), inactivating c-Jun N-terminal kinase (JNK), activating mitogen-activated protein kinase (MAPK), and inactivating phosphoinositide 3 kinase (PI3K) quercetin can regulate the production of pro-inflammatory cytokines (shown in Figure 6) in neurons [372]. 

Alzheimer’s disease is a chronic neurodegenerative disease, which results in the destruction of neurons in the cerebral cortex and some subcortical structures. Alzheimer’s disease is characterized by the appearance of senile wounds (made up of beta-amyloid (Aβ)). Aβ is a constituent part of a protein called amyloid precursor protein (APP), which plays an important role in the development, survival, and post-injury repair of neurons. As a result of the splitting of APP into smaller fragments under the influence of enzymes, Aβ is born, which can self-assemble into thick extracellular deposits known as senile/amyloid plaques [373]. In a study in mice, it was observed that i.p. administration of quercetin at doses of 25 mg/kg leads to decreased extracellular β-amyloidosis, tauopathy, astrogliosis, and microgliosis. It has also been observed to reduce the levels of paired helical filament (PHF), β-amyloid (βA) 1-40, and βA 1-42 and decreases the BACE1-mediated fragmentation of APP (shown in Figure 6) [373]. In other studies, it was observed that quercetin can inhibit acetylcholinesterase (AChE), but it has the role of breaking down acetylcholine in neuronal cells [308]. By encapsulating quercetin in nanoparticles, the bioavailability in the affected neurons and its ability to cross the blood–brain barrier was increased, countering the degenerative effects induced by Alzheimer’s disease. By inhibiting AChE, acetylcholine levels in synapses increase because the enzyme no longer can break down acetylcholine, which can improve signal transmission between neurons [374].

##### Antiosteoporosis Effect

Quercetin can inhibit bone resorption and promote bone formation [286]. The association of quercetin in pharmaceutical formulations with curcumin and polydatin can have a positive impact on health. This combination lowers the levels of two microRNAs (miR-21 and miR-146a) that play a role in inflammation. It also reduces the release of pro-inflammatory cytokines, promotes bone-building cell activity, and supports bone formation and mineralization. Additionally, it blocks the activation of certain proteins (p38 mitogen-activated protein kinase and nuclear factor-kappa B) that contribute to inflammation and bone breakdown. Overall, this combination helps reduce inflammation and improves bone health [375]. Lai et al., in an experiment on non-ovulating female rats, combined 2000 mg/kg of quercetin with 2400 IU/kg of vitamin D, 400 mg/kg of resveratrol, and 1040 mg/kg of genistein. This combination promoted an increase in bone mineral density and improved bone trabecular structure, leading to reduced bone loss associated with the postmenopausal stage [376]. 

##### Antihypertensive Effect 

According to scientific and clinical information on humans and laboratory animals, quercetin is currently considered a natural remedy. It improves mental and physical performance, reduces the risk of infections, eliminates oxygen free radicals, protects the body from chelation of metal ions, has an antiplatelet effect, and protects arteriolar microcirculation from ischemic lesions producing vasodilation [377,378,379,380].

**Figure 7:**
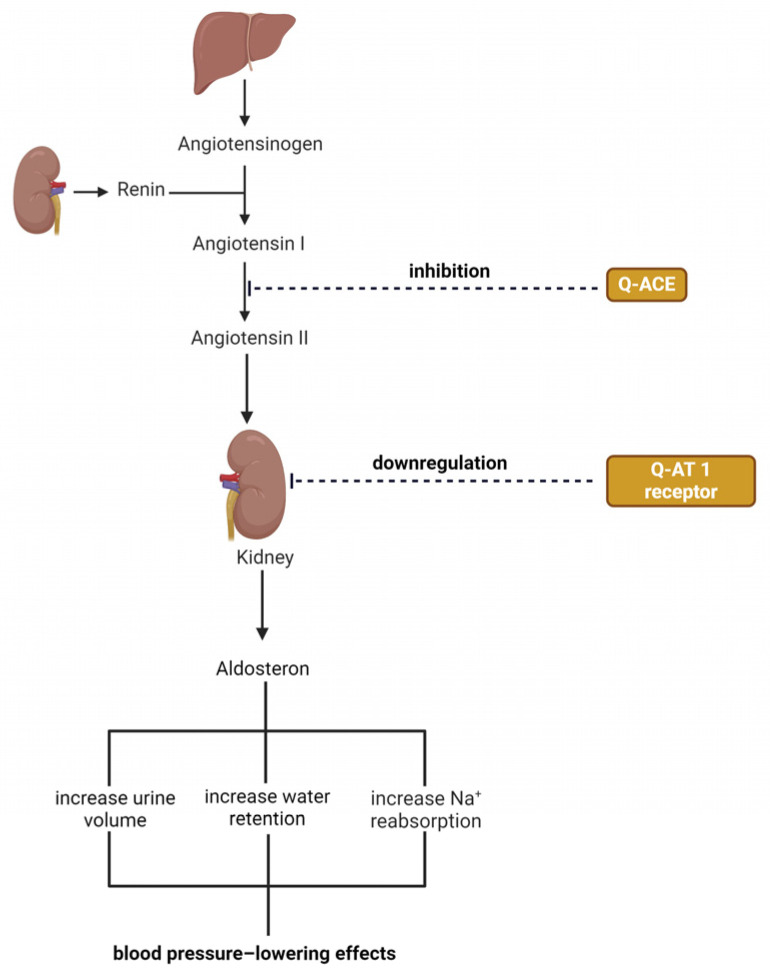
Possible mechanisms of action demonstrated in vitro and in vivo by which quercetin (Q) may interact with the renin–angiotensin–aldosterone system to lower blood pressure (scheme prepared with the Biorender). Legend: Q-ACE—quercetin inhibits the angiotensin-converting enzyme, Q-AT 1 receptor—quercetin downregulation the angiotensin 1 receptor.

Due to its vasodilator action and property to inhibit the production of 8-iso-prostaglandin F2α (a potent vasoconstrictor hormone), Abdelghaffar et al. observed in a study in hypertensive rats that quercetin can reduce systolic and diastolic blood pressure and can also decrease ventricular hypertrophy of renal and vascular lesions [381]. In the *British Journal of Nutrition*, it was shown that taking 730 mg/day of quercetin for 12 weeks resulted in a significant decrease in blood pressure in adults and overweight and obese people who had stage I hypertension [382]. Mackraj et al. compared the long-term antihypertensive effects of captopril with those of quercetin using rats. After 4 weeks, a significant decrease in blood pressure was observed, both in the groups treated with quercetin and in those treated with captopril. The decrease in blood pressure was also accompanied by the downregulation of the angiotensin-I receptor in the kidneys, an increase in urine volume, and an increase in urinary sodium excretion [383]. Hackl L.P.N et al. showed that quercetin can inhibit angiotensin-converting enzyme activity by modulating the cardiovascular response to angiotensin I (as shown in Figure 7). In addition, the researchers observed a 31% decrease in angiotensin-converting enzyme (ACE) activity after intravenous treatment of rats with quercetin (0.1 nmol/kg). These results suggest that quercetin may exhibit an ACE-inhibiting effect, similar to that of captopril, which explains some of its beneficial effects on blood pressure regulation [384].

##### Anti-Obesity Effect

Regarding obesity, studies in mice claim that quercetin has the ability to inhibit adipocyte differentiation and adipogenesis and that it can increase thermogenesis and improve lipolysis, which may favor increased energy expenditure. Recent findings indicate that quercetin enhances the expression of uncoupling protein 1 (UCP1) in brown adipose tissue (BAT), a key regulator of nonshivering thermogenesis. The activation of β3-adrenergic receptors (ADRB3) and the downstream signaling pathways, including protein kinase A (PKA) and AMP-activated protein kinase (AMPK), further supports the role of quercetin in promoting energy expenditure. Additionally, quercetin has been shown to increase the phosphorylation of hormone-sensitive lipase (HSL) and carnitine palmitoyltransferase 1 (CPT1α), facilitating lipid mobilization and oxidation. These mechanisms collectively contribute to enhanced metabolic activity and could provide a therapeutic strategy for managing obesity and related metabolic disorders [385].

Ahmed H. H. et al. studied the effects of quercetin and curcumin in the prevention of obesity by encapsulating them in PLGA nanoparticles with chitosan and polyethylene glycol (PEG). Following the treatment, a decrease in the weight of gonadal and subcutaneous adipose tissue, an improvement in the lipid profile, a reduction in serum levels of total cholesterol, triglycerides, and LDL, and an increase in HDL were observed. Decreases in glucose, insulin, and HOMA-IR (homeostatic model assessment of insulin resistance) levels, reduced levels of MDA (malondialdehyde), TLR4 (toll-like receptor 4), and NF-κB, as well as increased total antioxidant capacity [386], have also been observed.

##### Anticancer and Antitumor Effects

**Figure 8:**
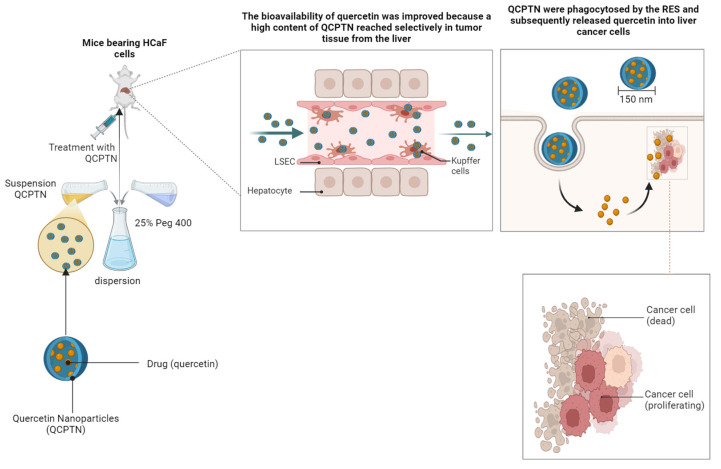
Study on the administration of quercetin-loaded PLGA-TPGS nanoparticles in targeted therapy for liver cancer (scheme prepared with the Biorender program). Legend: HCaF—Hepatocellular Carcinoma Fibroblast, QCPTN—quercetin-loaded PLGA-TPGS, Peg 400—polyethylene glycol 400, LSEC—Liver Sinusoidal Endothelial Cell, RES—Reticuloendothelial System.

Quercetin is also considered a good anticancer agent that can be used as a complement/alternative medication in the prevention and treatment of cancer [282]. Mirzaei et al. highlighted in vitro the antitumor effect of quercetin on PC-3 and LNCaP prostate cancer cell lines. The test results showed that quercetin significantly increased the rate of apoptosis in the PC-3 and LNCaP cell lines, inhibited cell proliferation by reducing the number of colonies, and exerted dose- and time-dependent cytotoxic effects. It was also observed that quercetin increased the rate of apoptotic cell death, which is beneficial in cancer-fighting studies, as apoptosis prevents cancer cells from multiplying and spreading. It affected the cell cycle of cancer cells, particularly their division and reproduction, in the sub-G1/G1 phase. Thus, quercetin prevented cancer cells from progressing to the S phase, where DNA replication occurs, from the G1 phase. Consequently, in the sub-G1 phase, programmed cell self-destruction (apoptosis) was induced. Another observation was that quercetin inhibited antiapoptotic pathways (the tumor progression biomarkers OPN a/b, KLK2, and the apoptosis-inducing marker P53), through which cancer cells protect themselves from apoptosis. All these results highlighted the antitumor effect of quercetin through the mechanism of inducing apoptosis and inhibiting antiapoptotic pathways [387]. Guan X. et al. tested the therapeutic efficacy of poly(lactate-co-glycolate) acid (PLGA) nanoparticles and D-alpha-tocopheryl polyethylene glycol succinate 1000 (TPGS) loaded with quercetin in liver cancer. The results obtained in vivo demonstrated that intravenous administration of quercetin nanoparticles was phagocytosed by the lysosomes of Kupffer cells in the liver (shown in Figure 8), releasing quercetin. After 0.5 h, this reached the tumor cells and reduced tumor development by 59.07%. The results obtained in vitro demonstrated that nanoparticles induced the death of HepG2 liver cancer cells in a concentration-dependent manner [388].

##### Antibacterial Effect

The antimicrobial effect of quercetin on Gram-positive and Gram-negative bacteria may be due to its ability to disintegrate the cell wall and cell membrane. Using transmission electron microscopy (TEM), it was observed that the damaged cell wall of *Escherichia coli* exhibited numerous structural abnormalities, such as its prominent lysis, cell distortion, and leakage of cytoplasmic materials. Also, the cytoplasmic membrane was separated from the cell wall, and the density of the endochylem was uneven. All these things led to further cell death [243]. Similarly, in *Staphylococcus aureus*, significant cell wall disruptions, thinning of cell membranes, chromatin lysis, leakage of endochylem contents, uneven endochylem density, shedding of extracellular pili, and nuclear cavitation were observed [389].

**Figure 9:**
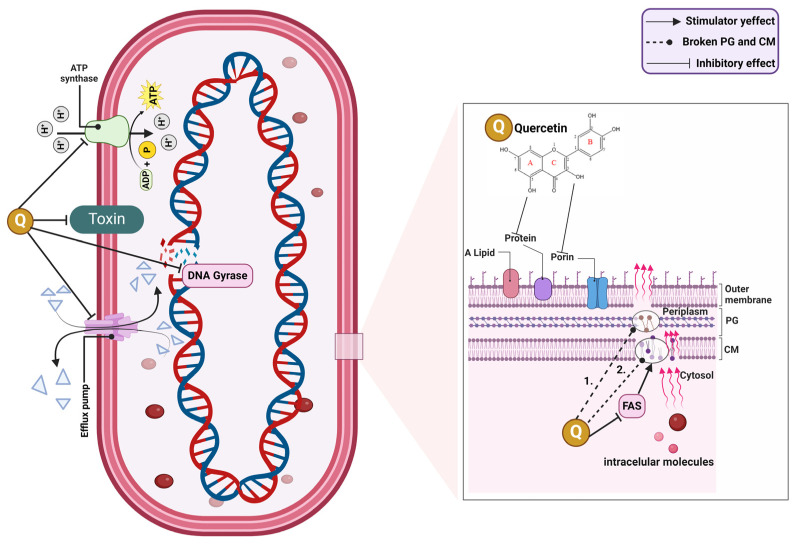
Quercetin’s mechanism action on Gram-positive and Gram-negative bacteria (image adapted from [283,284], prepared with Biorender). Legend: Q—quercetin, FAS—fatty acid synthase, PG—peptidoglycan, CM—cytoplasmic membrane, ATP—adenosine diphosphate, ADP—adenosine triphosphate.

Another mechanism of action of quercetin on bacteria is the ability to inhibit the synthesis of deoxyribonucleic acid by inhibiting bacterial DNA-gyrase, which ultimately leads to nucleoid and chromatin lysis. By blocking the ATP located in the membrane or cytoplasmic organelles, disturbances occur in the exchange of information, the active transport of compounds across the membrane, and the transfer of energy. By altering the permeability of the cell wall and inhibiting these enzymes, reproductive activity is blocked, developmental disruption occurs, and ultimately, bacterial cell death [390]. In other studies, quercetin in combination with various antibiotics produced an antibacterial effect on *Pseudomonas aeruginosa* manifested by disturbing the integrity of the cell wall, altering bacterial morphology, loss of porin, and inhibition of the efflux pump [391]. In *Klebsiella pneumoniae*, quercetin disrupts the structure and integrity of the cell wall [392]. According to the literature, quercetin exhibits several mechanisms of action that contribute to its antimicrobial properties. It can inhibit cell envelope synthesis by targeting fatty acid synthase (FAS) and disrupting peptidoglycan synthesis (1). Additionally, quercetin disrupts bacterial cell membranes (2) and interferes with nucleic acid synthesis by inhibiting DNA gyrase. Moreover, quercetin can inhibit bacterial virulence factors, including toxins. Its flavonoid nature allows it to inhibit efflux pumps, which can help reverse antimicrobial resistance and can also inhibit ATP synthase. These multifaceted actions underscore quercetin’s potential as an antimicrobial agent (Figure 9) [389,393] due to its flavonoid nature (Figure 9) [389,393].

##### Antifungal Effect 

Quercetin can be used in the treatment of fungal infections because, according to studies, it can induce apoptosis in fungal cells by affecting the functioning of mitochondria. It can inhibit the activity of proteolytic enzymes, and it can interfere with the adhesion mechanisms on the surface of the host cell, limiting the colonization of tissues by *Candida albicans*. Also, it can inhibit the formation of biofilm by sensitizing fungal cells to antifungal treatment [394]. Gao M. et al. showed that quercetin exhibits a strong synergic antifungal effect in combination with fluconazole against fluconazole-resistant *Candida albicans* isolates [395] or, if encapsulated, by electrospinning in PLGA—poly(D,L-lactide-co-glycolide)—and PCL—poly(ε-caprolactone)—nanofibers, without showing toxicity to HEK-293 (Human Embryonic Kidney 293) cells [396]. By introducing quercetin into liposomes alongside gallic acid, Giordani, B. et al. showed that quercetin’s antifungal effect on *Candida albicans* is much more pronounced than quercetin-only liposomes [397]. The minimum inhibitory concentration (MIC) of quercetin, found by Rocha et al., against the *Candida parapsilosis* complex consisting of *C. parapsilosis*, *C. orthopsilosis*, and *C. metapsilosis* was between 0.5 and 16 μg/mL. Among these strains, the antifungal effect of quercetin was more pronounced on *C. metapsilosis* biofilms, according to CLSM (confocal laser scanning microscopy) analysis, in terms of biomass, roughness coefficient, maximum thickness, and thickness of the entire area [398]. 

##### Antiviral Effect

Numerous in vivo and in vitro studies have highlighted the antiviral properties of quercetin against a wide range of viruses such as influenza A virus [399], SARS-CoV-2 [400], hepatitis B virus [401], Ebola virus *(Zair ebolavirus species)* [402], varicella-zoster virus and human cytomegalovirus [403], hepatitis C virus, herpes simplex types 1 and 2, human immunodeficiency virus (HIV) [309], etc. The antiviral effects of quercetin are given by the ability to inhibit viral polymerase enzymes, reverse transcriptase of HIV, viral proteases, and DNA gyrase of the herpes simplex virus [309]. Dias S. et al. highlighted the antiviral effect of quercetin against the hepatitis C virus (HCV). The results suggested that quercetin interacts with multiple stages of the HCV life cycle, inhibiting both replication and spread of infection. Thus, in HCV-infected Huh-7.5 liver cells, quercetin reduced the viral load by up to 85%, and in human primary hepatocytes (PHH) by up to 92% and decreased the ability to spread infection by 65% [25].

#### 3.3.4. Safety, Dosage, and Drug Interactions

The average intake of aglyconic flavonoids without tearubigens is considered to be between 150 and 600 mg/day worldwide [404], and the average dietary intake of quercetin is considered to be between 4.37 and 18.48 mg/day. However, the values of this intake may vary from country to country depending on gender and age [239].

The intake of quercetin based on the consumption of fruits and vegetables can vary between 5 and 100 mg/day depending on their type and the amount consumed. In the case of high consumption of foods rich in quercetin (onions or apples), the intake can be 5 times higher. Studies claim that the consumption of fats and foods rich in pectins, lecithins, and oligosaccharides increases the absorption of quercetin in the body. In clinical trials, quercetin is used in a concentration of 500–1000 mg/day, in divided doses, and as a dietary supplement in doses of 50 mg, 100 mg, and 500 mg [308]. 

The recommended dose of quercetin for adults is between 100 and 250 mg taken three times a day but can vary from region to region [253]. It is contraindicated for use by pregnant, breastfeeding women and people with kidney disease. Studies have shown that quercetin may interact with ciprofloxacin or levofloxacin due to affinity for bacterial DNA-gyrase situs. Therefore, due to the same site of action, it can act as a competitive inhibitor of quinolone antibiotics and therefore decrease their efficacy [307]. Other studies show that quercetin is a potent inhibitor of CYP3A4 and therefore may increase serum concentrations of drugs metabolized by this enzyme (statins, certain antidepressants) and their toxicity [308,405]. It can also affect the pharmacokinetics of drugs that are substrates for P-glycoprotein (e.g., immunosuppressants) [405].

## 4. Conclusions

Quercetin is a safe, natural antioxidant with a flavonol chemical structure and the ability to eliminate free radicals and combat oxidative stress. By manifesting this effect, quercetin may be a key in combating age-related diseases and other conditions influenced by cell damage. In addition, by inhibiting cytokines and inflammatory enzymes, it paves the way for application in the treatment of inflammatory conditions such as asthma or allergies. It can also prevent the proliferation of cancer cells and trigger apoptosis in various types of cancer, including liver, breast, prostate cancer, etc. Although studies have highlighted some beneficial effects of quercetin, its absorption, solubility, and poor stability can negatively influence its bioavailability in the body. Thus, researchers have succeeded in various innovative strategies to improve the bioactivity of quercetin by encapsulation in systems (liposomes, emulsions, or suspensions) or by complexation with cyclodextrins. According to the data provided in this paper, we believe that quercetin has the potential to become a valuable therapeutic agent in modern medicine.

## Figures and Tables

**Table 1:** Natural sources of flavones and their bioactivity.

Name of the Flavone	Bioactivities	Natural Sources	Ref.
Apigenin	Anti-inflammatory, antibacterial, antiviral, antiallergic, neuroprotective, sedative, antispasmodic, anticancer	*Daphne genkwa* Siebold & Zucc., *Alnus glutinosa* (L.) Gaertn., *Hypericum perforatum* L., *Matricaria chamomilla* L., *Petroselinum crispum* (Mill.) Fuss, Apium graveolens L., *Crataegus monogyna* Jacq., *Passiflora incarnata* L., *Equisetum arvense* L., *Populus nigra* L., *Zea mays* L.	[11,44,49,50,51,52,53,54]
Acacetin	Antiatherosclerosis, anti-ischemic	*Ziziphora clinopodioides* Lam., *Agastache rugosa* (Fisch. & C.A.Mey.) Kuntze, *Cydonia oblonga* Mill, *Petroselinum crispum* (Mill.) Fuss	[11,55,56,57,58]
Sinensetin	Anti-inflammatory, antioxidant, antimicrobial, anti-obesity, neuroprotective, venotonic	*Orthosiphon aristatus* (Blume) Miq., *Orthosiphon stamineus* Benth, *Citrus reticulata* Blanco	[59,60]
Nobiletin	Anti-inflammatory, antitumor, antiviral, antioxidant, anticancer, neuroprotective	*Citrus tangerina* Tanaka, *Citrus erythrosa* hort. ex Tanaka, *Citrus depressa* Hayata, *Citrus sinensis* (L.) Osbeck, *Citrus limon* (L.) Osbeck, *Laurus nobilis* L., *Physalis alkekengi* L.	[61,62,63,64]
Vitexin	Antiepileptic, neuroprotective, antitumor action against gastric cancer, cardioprotective	*Crataegus monogyna* Hayek, *Passiflora incarnata* L.	[46,65,66,67,68]
Luteolin	Anticancer, antiviral, anti-inflammatory, antioxidant, neuroprotective	*Passiflora incarnata* L., *Equisetum arvense* L., *Zea mays* L.	[11,69,70,71]
Casticin	Antiallergic, anti-inflammatory, anticancer	*Vitex agnus-castus* L., *Vitex rotundifolia* L.f.	[72,73,74]
Artemetin	Hypotensive, inhibition of hyperpigmentation, antioxidant, hepatoprotective	*Artemisia absinthium* L., *Achillea millefolium* L., *Vitex glabrata* R.Br.	[75,76,77,78]
Diosmin	Antioxidant, anti-inflammatory, cicatrizing, antiplatelet, hypolipidemic, antiatherosclerosis, vasoprotective, venotonic, antiedematous	*Citrus sinensis* (L.) Osbeck, *Citrus limon* (L.) Osbeck, *Citrus reticulata* Blanco, *Citrus bergamia* Risso, *Hyssopus officinalis* L.	[79,80,81,82,83,84,85,86,87]

**Table 2:** Natural sources of flavonols and their bioactivity.

Name of the Flavonol	Bioactivities	Natural Sources	Ref.
Hybiscetol	Neuroprotective	*Hibiscus sabdariffa* L.	[97,98,99]
Kaempferol	Antibacterial, antifungal, antiprotozoal, anti-inflammatory, anticancer	*Crataegus monogyna* Hayek, *Ginkgo biloba* L., *Betula pendula* Roth, *Passiflora incarnata* L., *Equisetum arvense* L., *Populus nigra* L., *Sophora japonica* L., *Solidago virgaurea* L., *Vaccinium macrocarpon* Aiton, *Vitis vinifera* L.	[11,100,101,102]
Myricetol	Wound healing, antioxidant, antiatherosclerosis	*Pistacia lentiscus L.*, *Myrica rubra* (Lour.) Siebold & Zucc., *Lycium barbarum* L., *Vigna subterranea* (L.) Verdc., *Acacia confusa* Merr., *Trifolium repens* L.	[103,104,105]
Rutin	Antioxidant, anti-inflammatory, neuroprotective, antigout effects, cytoprotective, vasoprotective, anticancer	*Sambucus nigra* L., *Sophora japonica* L. (*Sophorae flos immaturus*—content over 18%), *Solidago virgaurea* L., *Fagopyrum esculentum* Moench—3.42% content, *Ruta graveolens* L, *Hypericum perforatum* L.	[91,92,106,107,108,109,110,111]
Isorhamnetin	Cardioprotective, anticancer, hepatoprotective, lipid-lowering, anti-inflammatory	*Hippophae rhamnoides L*., *Ginkgo biloba L.*, *Bupleurum chinense* DC., *Vaccinium myrtillus* L., *Sambucus nigra* L., *Calendula officinalis* L.	[94,112,113,114]
Quercetin	Anti-inflammatory, antioxidant, antiapoptotic, antidiabetic, antihypertensive, cardioprotective, neuroprotective, antiviral, antibacterial, hepatoprotective, anticancer	*Crataegus monogyna* Hayek, *Ginkgo biloba* L., *Betula pendula* Roth, *Equisetum arvense* L., *Populus nigra* L., *Sophora japonica* L., *Solidago virgaurea* L., *Vaccinium macrocarpon* Aiton, *Vitis vinifera* L., *Hypericum perforatum* L., *Sambucus canadensis* L.	[11,115,116,117]
Morin	Anti-inflammatory, antioxidant, antiapoptotic, neuroprotective	*Maclura tinctoria* (L.) D.Don ex Steud., *Maclura pomifera* (Raf.) C.K.Schneid., *Psidium guajava L.*	[95,118]

**Table 3:** Natural sources of flavanones and their bioactivity.

Name of the Flavanone	Bioactivities	Natural Sources	Ref.
Naringenin	Cardioprotective, anticancer, hepatoprotective, hypolipidemic	*Citrus sinensis* (L.) Osbeck (aurantii amari epicarpium et mesocarpium), *Citrus paradisi* Macfad., *Citrus bergamia* Risso, *Arabidopsis thaliana* (L.) Heynh., *Lippia graveolens* Kunth	[120,127,128,129,130]
Naringin	Antitussive, expectorant, anti-inflammatory, neuroprotective	*Citrus sinensis* (L.) Osbeck (aurantii amari epicarpium et mesocarpium), *Citrus paradisi* Macfad., *Citrus bergamia* Risso, Citrus grandis Osbeck	[131,132,133,134,135]
Eriodyctiol	Hypolipidemic, antioxidant, anti-inflammatory, anticancer	*Eriodictyon californicum* (Hook. & Arn.) Torr., *Rosa canina* L., Ficus sagittifolia Warb. ex Mildbr. & Burret	[122,136,137,138,139]
Hesperetin	Anticancer, hepatoprotective, anti-hyperuricemic, anti-inflammatory	*Citrus limon* (L.) Osbeck, *Citrus paradisi* Macfad., *Citrus sinensis* (L.) Osbeck	[126,140,141,142]
Hesperidin	Anticancer, antidepressant	*Citrus limon* (L.) Osbeck, *Citrus paradisi* Macfad., *Citrus sinensis* (L.) Osbeck, *Citrus reticulata* Blanco	[142,143,144,145]
Pinocembrin	Cardioprotective, wound healing, anti-inflammatory, antioxidant, antiapoptotic, antifibrotic	*Populus nigra* L., Alpinia officinarum Hance, *Eriodictyon californicum* (Hook. & Arn.) Torr., *Glycyrrhiza glabra* L., *Piper sarmentosum* Roxb.	[146,147,148,149,150]
Taxifolin	Antidiabetic	*Pinus densiflora* Siebold & Zucc., Larix gmelinii (Rupr.) Kuzen., *Cedrus deodara* (Roxb. ex D.Don) G.Don, *Silybum marianum* (L.) Gaertn., *Abies nephrolepis* (Trautv. ex Maxim.) Maxim.	[151,152,153,154]

**Table 4:** Natural sources of proanthocyanidins and their bioactivity.

Name of the Proantocyanidol	Bioactivities	Natural Sources	Ref.	
Epicatechin	Antioxidant, anticancer, hepatoprotective	*Campomanesia adamantium* (Cambess.) O. Berg, *Camellia sinensis* (L.) Kuntze, Fallopia japonica Houtt, *Fagopyrum esculentum* Moench	[163,164,165,166,167,168]	
Gallocatechin	Wound healing, anti-inflammatory, neuroprotective, antioxidant	*Camellia sinensis* (L.) Kuntze, *Punica granatum* L., *Eugenia brasiliensis* Lam.	[169,170,171,172]	
Epigallocatechin	Antiviral, antifungal, antibacterial	*Camellia sinensis* (L.) Kuntze	[173,174]	

**Table 6:** Natural sources of chalcones and their bioactivity.

Name of the Chalcone	Bioactivities	Natural Sources	Ref.
Isoliquiritigenin	Anticancer, anti-inflammatory	*Glycyrrhiza glabra* L., *Genista tinctoria* L., *Cissus polyantha* Gilg & M. Brandt	[214,216,217,218,219,220]
Xanthohumol	Anticancer, hepatoprotective	*Humulus lupulus* L.	[221,222,223]
Phloretin	Antifungal, antidiabetic nephropathy, anti-multiple sclerosis	*Pieris japonica* (Thunb.) D. Don ex G. Don, *Hovenia dulcis* Thunb	[206,224,225,226,227]
Butein	Antiulcer, anti-inflammatory, anticancer, antioxidant, antimicrobial	*Rhus verniciflua* Stokes, *Butea monosperma* (Lam.) Kuntze, *Dalbergia odorifera* T.C. Chen	[228,229,230,231,232]

**Table 7:** Natural sources of quercetin.

Sources	Amount of Q	Extraction Solvent	Detection Technique	Ref.
*Ginkgo biloba* L. (folium)	3.40 ± 0.11 mg/g	Ethanol	HPLC	[248]
*Morus alba* L. (folium)	6.29 ± 1.20 mg/g	Ethanol	HPLC	[248]
*Phyllanthus emblica* L.	10.20 ± 1.04 mg/g	Ethanol	HPLC	[248]
	5.816 ± 2.81 mg/g	Ethanol	UV-VIS	[249]
*Hypericum perforatum* L.	29.48 ± 1.3 μg/mL	Ethyl acetate	HPLC	[250]
*Hypericum perforatum* L. (folium)	61.8 ± 1.3 mg/g	Acetone	HPLC	[251]
*Sambucus nigra* L.	28.214 mg/g	Water	HPLC	[252]
*Lactuca crispa* (L.) Roth	30.6 mg (100 g)^−1^	Ethanol/water/ HCI (50:20:8)	HPLC	[253]
*Camellia sinensis* (L.) Kuntze	1070.0 ± 0.09 mg/kg	Aqueous methanol solution	HPLC	[254]
*Allium cepa* L.	457 ± 3.75 mg/g	Ethyl acetate	HPLC	[255]
*Capsicum annuum* L.	66.0 ± 1.6 mg/100 g	80% ethanol	HPLC	[256]
	35.3 ± 1.1 mg/100 g	80% ethanol	HPLC	[256]
*Hibiscus esculentus* L.	205.5 ± 0.05 mg/kg	Aqueous methanol solution	HPLC	[254]
*Moringa oleifera* Lam.	232.5 ± 0.02 mg/kg	Aqueous methanol solution	HPLC	[254]
*Allium fistulosum* L. (folium)	95 ± 3 μg/mL	30% ethanol	HPLC	[257]
*Centella asiatica* (L.) Urb. (folium et radix)	2501.1 ± 6.1 mg/kg	Methanol	HPLC	[258]
*Hypericum hircinum* L.	0.44 mg/g	70% ethanol	HPLC	[259]
*Nasturtium officinale* R.Br. (flos)	1459.30 ± 12.95 ng/g	80% methanol	UPLC-MS	[260]
*Brassica botrytis* Mill.	4.11 ± 0.3 mg/100 g	Ethanol	HPLC	[261]
Brassica *capitata* DC. ex H.Lév.	11.92 ± 0.72 mg/100 g	Ethanol	HPLC	[261]
*Apium graveolens* L.	4.95 ± 0.78 mg/kg	Ethanol 96%	HPLC	[262]
*Calendula officinalis* L.	17.42 ± 1.55 mg/kg	Ethanol 96%	HPLC	[262]
*Coriandrum sativum* L.	339.5 ± 6.28 mg/kg	Methanol	HPLC	[263]
*Capparis spinosa* L. (flos)	128.0 μg/g	Methanol/acetic acid/water (100:2:100)	LC-ESI/QTrap/MS/MS	[264]
*Capparis orientalis* Veill. (flore gemmae)	908.0 μg/g	Methanol/acetic acid/water (100:2:100)	LC-ESI/QTrap/MS/MS	[264]
*Asparagus acutifolius* L. (pericarpium)	9.08 ± 0.20 mg/kg	Ethanol/water (80:20)	HPLC	[265]
*Prunus domestica* L. (folium)	311.13 ± 3.14 mg/100 g	30% ethanol and 6N HCI	HPLC	[266]
*Malus domestica* Baumg.	7445.32 ± 29.25 mg/100 g	Methanol and 1.5 M HCI	HPLC	[267]
*Vaccinium myrtillus* L.	11.35 ± 1.84 mg/kg	Methanol/formic acid (99.5/0.5)	HPLC	[268]

**Table 8:** Types of glycosidic quercetin found in nature.

Chemical Structure	Glycaride/Glucuronide Bound to Aglycone Quercetin	Sources	Amount	Name of the Quercetin Derivative	Refs.
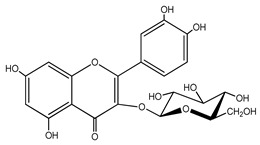	Glucose	*Hibiscus mutabilis* L.	0.25–8 μg/mL	Quercetin 3-O-glucozide/isoquercitrin	[238,269,270] [271] [272]
		*Morus alba* L.	972.48 ± 0.014 mg/100 g DW		
		*Allium cepa* L.	0.49 μmol/g DW		
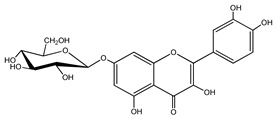	Glucose	*Chrysanthemum segetum* L., *Asclepias syriaca* L., *Carthamus tinctorius* L., *Brasenia schreberi* J.F. Gmel.		Quercetina 7-O-β-D- glucopyranoside (Q7G)	[34,238,273,274,275,276]
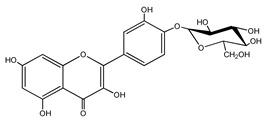	Glucose	*Allium cepa* L.	4.33 μmol/g DW	Quercetin 4’-O-glucoside/spiraeoside	[276,277]
		*Allium cepa* var. *roseum* Alef.	41.39 mg/g DW)		
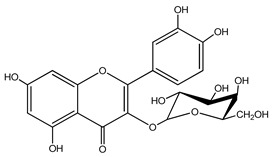	Galactose	*Solidago virgaurea* L.	24.160 μg/mL	Quercetin 3-O-galactozide/hyperozide	[238,269,270] [271]
		*Hypericum perforatum* L.	665.38 mg %		
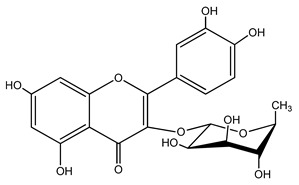	Rhamnose	*Fagopyrum tataricum* (L.) Gaertn.	0.090 ± 0.019% DW	Quercetin-3-O-rhamnoside/quercitrin	[272]
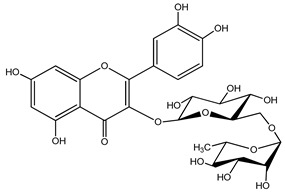	Rhamnose and glucose	*Morus alba* L.	194.3 ± 0.54 mg/100 g DW	Quercetin 3-O-rhamnosil-glucoside/rutoside/rutin	[34,238,273,274,275,276]
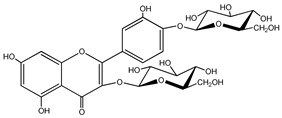	Two glucose molecules	*Allium cepa* L.	4.66 μmol/g DW	Quercetin 3,4’-diglucoside	[278]
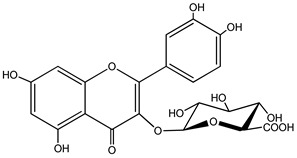	Glucuronic acid	*Phaseolus vulgaris* L., *Cichorium intybus* L.	-	Quercetin 3-O-glucuronide/Quercituron	[279,280,281]

The chemical structure of quercetin glycoside found in nature is prepared with Biorender software.

**Table 9:** Physicochemical properties of quercetin.

Chemical Structure of Quercetin	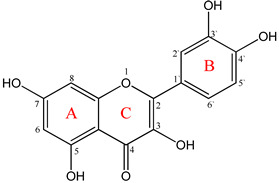	
Physical Constants	Values	Ref.
Appearance	It is a solid substance, which comes in the form of a fine crystalline powder, yellow to greenish in color	[233,245,284,305]
Melting point	313–316.5 °C	
Water solubility	0.001 mg/mL at 25 °C or 60 mg/mL at 16 °C	
Highly soluble	In ether, methanol	
Soluble	In acetone, pyridine, glacial acetic acid, aqueous alkaline solutions, ethyl acetate	
Hardly soluble	In ether, benzene, chloroform	
Density	1.799 g/cm^3^	
Stability	Affected by pH, temperature, storage time, oxygen, metal ions	

Chemical structure of quercetin is prepared with Biorender software.

**Table 10:** Ways to increase the bioavailability of quercetin.

Preparation Technique	Advantages	Substances Used	PF Obtained	Ref.
Ethanol nano precipitation	The particle sizes were around 200 nm. Manifestation of a low degree of toxicity to normal MRC-5 cells of human pulmonary fibroblasts. Manifested anticancer effect against MDA-MB-231.	QUE, βCD, Ethanol absolute	INC	[345]
Electrospinning	Manifestation of bacteriostatic effects against *Staphylococcus aureus* and *Escherichia coli.*	QUE, methanol, βCD, zeina, DMF	Nanofilms	[346]
Stirring	Increased solubility and stability of QUE and RSV. Sizes between 82 and 103 nm. The formulations were biocompatible with human corneal and conjunctival epithelial cells. Combating the feeling of dry eyes.	QUE, HPβCD	Binary and ternary ICs	[347]
Stirring, freeze-drying, spinning	Antibacterial effect manifested against *Escherichia coli.* Improvement in the physicochemical characteristics of QUE.	QUE, NaBH_4_, oleic acid AgNO_3_, anhydrous βCD	ICs–AgNPs	[348]
Supercritical antisolvent precipitation	Increased solubility of QUE in water, increased therapeutic efficiency, high deposition of QUE in the lungs, and combating the symptoms of acute respiratory distress syndrome.	QUE, βCD, DMSO CO_2_, methanol	Ms	[349]
Thin film hydration	Antitumor effect manifested against HepG2 cells, high EE (98.63 ± 1.28%), and sustained release.	Folic acid, chitosan	LP	[350]
Green ethanol injection	EE was 96.9%, showed anticancer effect against PC3 cells of PC, reduced ability to form spheroids, and inhibited cancer cell migration.	QUE, HA, cholesterol TPGS, DOTAP	NLP	[351]
Solvent injection	Reduced the severity of Co-Amox-induced liver damage, regulated transcription factors (SIRT1 and Nrf 2), and prevented intestinal dysbiosis.	QUE, Phosopholipon 90 G, cholesterol, Ethanol absolute	NLP	[352]
Thin-film hydration	Anticancer effect manifested against breast adenocarcinoma (MCF-7) and lung adenocarcinoma (A549).	QUE, cholesterol, gallic acid	NLP	[353]
Solvent and antisolvent precipitation	Anticancer effect against alveolar epithelial cells (A549), manifested by increasing Bax expression, inducing apoptosis, reducing ATP, and generating ROS.	QUE, Cur, ethanol, Pluronic F-127, PVA	NPs	[354]
Nanoprecipitation	Targeted release of QUE into prostate cancer cells.	PLGA, DMSO, PVA, QUE, FA, chitosan	NPs	[355]
Ionic gelation	Sustained release for 36 h, induction of cytotoxicity, arrest of G0/G1 cell cycle, and apoptosis in HCT116 cancer cells.	QUE, 5-fluorouracil, chitosan, TPP-Na	NPs	[356]
Ionic gelation	Uniform and homogeneous distribution of active compounds in NPs obtained. The average size of NPs was 200 μm. Combating oxidative stress induced by H_2_O_2_ in human neuroblastoma cells SH-SY5Y.	QUE, VPA, chitosan, TPP-Na	NPs	[357]
Conjugation	Cytotoxic effect manifested towards HeLa and Caco-2 cancer cell lines, targeted delivery of active compounds at the nuclear or perinuclear level, increases hydrophilicity, improvement in the pharmacokinetic profile of QUE and HA.	QUE, HA, DCC, DMAP, AgNO_3_, PEG, FA	PF-AgNPs	[358]
Complex coacervation	QUE was trapped in the Ms matrix according to SEM analysis and protected from degradation. It showed a high antibacterial and antifungal effect against *Escherichia coli* and *Candida albicans*.	QUE, chitosan, sodium alginate, calcium chloride	Ms	[359]
Complex coacervation	High EE, over 86.07%, prolonged release, for 24 h, at the intestinal level, stable in the physiological environment, presented a similar appearance and a compact structure.	QUE, chitosan, sodium alginate, calcium chloride	Ms	[360]
Soaking and stirring	Improved QUE release processes, stability, and achieving optimal release concentration.	TETA, NH_2_, PLL, QUE	MSN	[361]

Legend: PF—pharmaceutical form, βCD—β-cyclodextrin, INCs—inclusion nanocomplexes, DMF—dimethyl formamide, QUE—quercetin, RSV—resveratrol, HA—hyaluronic acid, HPβCD—Hydroxypropyl-β-cyclodextrin, NaBH_4_—sodium tetrahydro borate, AgNO_3_—silver nitrate, ICs—inclusion complexes, ICs-AgNPs—ternary inclusion complexes consisting of β-CD nanosponges, silver and quercetin nanoparticles, EE—Entrapping efficiency, DOTAP—1,2-Dioleoyl-3-dimethylammonium propane, TPGS—D-α-tocopherol polyethylene glycol succinate, PC—prostate cancer, Co-amox—amoxicillin/clavulanate, LPs—liposomes, QUE-GA-NLP—quercetin and gallic acid nanoliposomes, Cur—curcumin, PVA—polyvinyl alcohol, NPs—nanoparticles, Bax—Proapoptotic protein, ATP—adenosine triphosphate, ROS—reactive oxygen species, PLGA—Poly(D,L-lactide-co-glycolide, DMSO—dimethyl sulfoxide, FA—folic acid, TPP-Na—sodium tripolyphosphate, VPA—valproic acid, H_2_O_2_—hydrogen peroxide, DCC—1, 3-dicyclohexylcarbodiimide, DMAP—4 dimethyl aminopyridine, PEG—polyethylene glycol, PF-AgNPs—silver nanoparticles conjugated with hyaluronic acid and quercetin coated with polyethylene glycol and folic acid, TETA—triethylenetetramine, MSN—mesoporous silica nanoparticles, NH_2_—aminopropyl-triethoxysilane, PLL—poly-L-lysine.

## Data Availability

No new data were created or analyzed in this study.
